# The posteroventral part of the medial amygdala nucleus glutamatergic neurons encodes conspecifics’ individual identity in rodents

**DOI:** 10.1126/sciadv.ady9830

**Published:** 2026-05-22

**Authors:** Lu Zheng, Libiao Pan, Xiaoyu Fu, Siyu Wang (王思羽), Yue Wu, Hanyang Xiao, Jiachao Yang, Siyu Wang (王思雨), Li Yang, Xiaotong Wu, Fada Pan, Hongbin Yang, Gao Chen, Hao Wang

**Affiliations:** ^1^ Department of Neurosurgery of Second Affiliated Hospital and School of Brain Science and Brain Medicine, Zhejiang Key Laboratory of Research and Transformation for Major Neurosurgical Diseases, Key Laboratory for Biomedical Engineering of Education Ministry, Zhejiang University School of Medicine, Hangzhou 310058, Zhejiang, China.; ^2^ Nanhu Brain-computer Interface Institute, Hangzhou 311100, China.; ^3^ NHC and CAMS Key Laboratory of Medical Neurobiology, MOE Frontier Science Center for Brain Research and Brain Machine Integration, Key Laboratory of Precise Treatment and Clinical Translational Research of Neurological Diseases, Zhejiang University, Hangzhou 310058, Zhejiang, China.; ^4^ Liangzhu Laboratory, State Key Laboratory of Brain-Machine Intelligence, Zhejiang University, Hangzhou 311121, China.; ^5^ College of Forensic Science, Key Laboratory of National Health Commission for Forensic Science, National Biosafety Evidence Foundation, Xi’an Jiaotong University, Xi’an 710061, Shaanxi, China.; ^6^ Lingang Laboratory, Shanghai 200031, China.

## Abstract

The medial amygdala (MeA) processes social olfactory cues, but its precise neural mechanisms remain unclear. We identified the posteroventral MeA (MeApv) as critical for individual conspecific odor discrimination in mice. Exposure to conspecifics or their odors markedly elevates calcium signals and c-Fos expression in MeApv VGluT2-positive neurons. Optogenetic silencing of these neurons or activating Gad2-positive neurons disrupts odor-driven social behaviors, including identity recognition, odor discrimination, and sex discrimination. Social information is directly relayed from the accessory olfactory bulb (AOB) to the MeApv, and acute AOB-MeApv pathway disruption impairs social discrimination. A distinct MeApv VGluT2-positive neuron population encodes individual-specific cues, as revealed by microendoscopic calcium imaging at a single-cell resolution. Selective silencing of these neurons induces deficits in odor-guided social interactions with related conspecifics, confirming the MeApv as a central hub for social information encoding. These findings establish the MeApv’s dual necessity and sufficiency in translating olfactory signals into social behavioral responses.

## Teaser

The MeApv is a neural hub that decodes social scents to recognize individuals.

## INTRODUCTION

Appropriate social behavior forms the foundation of social networks, which are crucial for survival and health in humans and other animals (*1*). Effective social interactions rely on the ability to detect and recognize conspecific cues, guiding complex behaviors such as aggression, avoidance, cooperation, and mating (*2*–*4*). Socially relevant information—including gender, reproductive status, identity, social hierarchy, and health—is encoded by different sensory systems. In humans, visual cues (e.g., facial features and body posture) and auditory cues (e.g., voice characteristics) are essential for social behavior. In rodents, however, initiating social behavior depends primarily on olfactory cues that convey reproductive status, dominance, and familiarity through pheromones (*3*–*7*).

Recent studies in rodents suggest that the brain processes olfactory social cues through specialized neural circuits distinct from those involved in nonsocial information. Nonsocial cues, such as those related to food and the environment, are mainly processed by the main olfactory system, which includes the main olfactory epithelium and the main olfactory bulb (MOB). In contrast, social olfactory cues engage both the main and accessory olfactory systems, with their information converging in higher brain regions, such as the medial amygdala (MeA), ventromedial hypothalamus, and bed nucleus of the stria terminalis (*8*–*11*).

Structurally, as a subdivision of the amygdalar complex, the MeA is composed of the anteroventral (MeAav), anterodorsal (MeAad), posteroventral (MeApv), and posterodorsal (MeApd) nuclei. Functionally, the MeA is a key site where chemosensory information from both olfactory systems integrates (*12*). In the main olfactory system, sensory input flows from the MOB to the olfactory amygdala, piriform cortex, and anterior and posterolateral cortical nuclei before projecting to the MeA. In the accessory olfactory system, vomeronasal information travels through the accessory olfactory bulb (AOB) to specific MeA regions, as well as the posteromedial cortical nucleus. Retrograde labeling studies show that the MeAad, MeAav, and MeApv receive direct inputs from the MOB, while the MeApd is prominently innervated by the AOB (*13*–*16*). Although the MeA plays a pivotal role in processing olfactory social signals, its precise role in converting social odors into behaviorally relevant outputs remains unclear.

In this study, we investigated how circuits in the posterior MeA, particularly the MeApv, regulate social responses to conspecific cues. We found that vesicular glutamate transporter 2 (VGluT2)–positive neurons in the MeApv were selectively activated by socially relevant odors. Optogenetic silencing of these neurons, or activation of glutamate decarboxylase 2 (Gad2)–positive neurons, impaired odor-dependent social behaviors. Using microendoscopic calcium imaging and activity-dependent labeling combined with optogenetics, we identified a subset of MeApv VGluT2-positive neurons that encode the identity of individual conspecifics. The activity of these neurons was both necessary and sufficient to modulate social behavior.

## RESULTS

### MeApv VGluT2 neurons are activated during exploration of social information

**Fig. 1.**
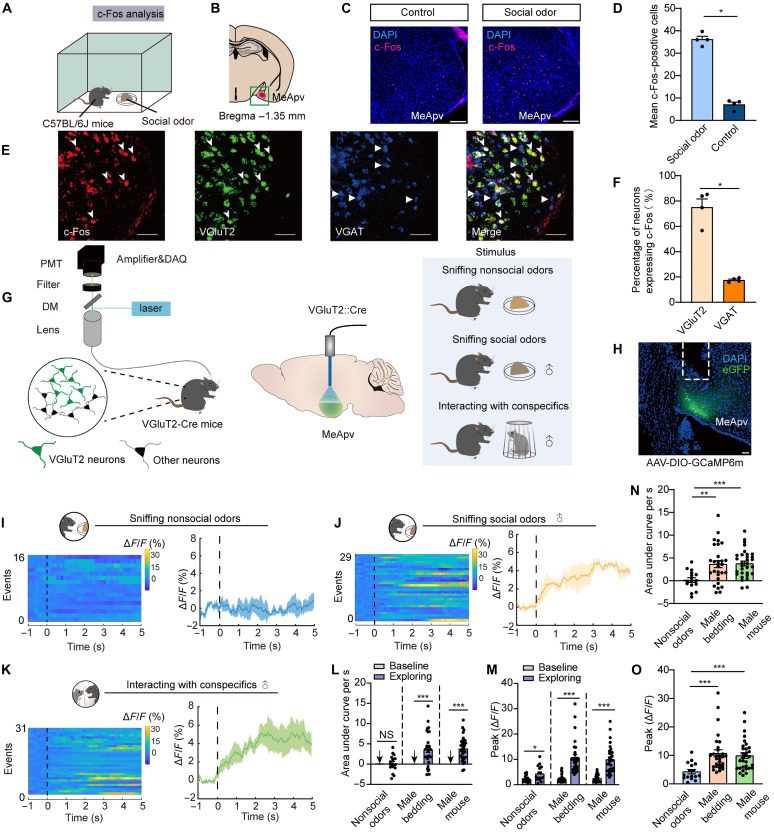
MeApv VGluT2 neurons are highly activated during social exploration. (**A**) Experimental schematic for c-Fos analysis following social interaction. (**B** and **C**) Representative images showing c-Fos expression in the MeApv. Scale bars, 80 μm. (**D**) Quantification of c-Fos^+^ cells (*n* = 4 mice per group; *P* = 0.0286, Mann-Whitney test). (**E**) Representative images of c-Fos, VGluT2, and VGAT expression and merge image. Scale bars, 50 μm. (**F**) Quantification of the numbers of cells expressing c-Fos (*n* = 4 mice per group; *P* = 0.0286, Mann-Whitney test). (**G**) Schematic diagram of calcium signal recording. (**H**) Schematic of AAV injection and fiber optic implantation in VGluT2-Cre mice. Scale bar, 50 μm. (**I** to **K**) Normalized GCaMP6m activity (heatmaps and mean signals) during (I) nonsocial odor exposure, (J) social odor exposure, and (K) social interaction. (**L** and **N**) AUC per second of calcium signals measured in exposure to social/nonsocial cues. (L) For nonsocial odors, *n* = 16 events from three mice, *P* = 0.7489, unpaired *t* test; for social odors, *n* = 29 events from three mice, *P* < 0.0001, unpaired *t* test; for male mice, *n* = 31 events from three mice, *P* < 0.0001, unpaired *t* test; (N) nonsocial odors versus social odors, *P* = 0.0031; nonsocial odors versus male mice, *P* < 0.0001, unpaired *t* test. (**M** and **O**) Peak of GCaMP6m signals. (M) For nonsocial odors, *n* = 16 events from three mice, *P* = 0.0234, Mann-Whitney test; for social odors, *n* = 29 events from three mice, *P* < 0.0001, Mann-Whitney test; for male mice, *n* = 31 events from three mice, *P* < 0.0001, Mann-Whitney test; (O) nonsocial odors versus social odors, *P* < 0.0001; nonsocial odors versus male mice, *P* < 0.0001, Mann-Whitney test. *P < 0.05; **P < 0.01; ***P < 0.001; NS, not significant. Error bar, SEM.

To explore whether the MeApv is involved in processing social olfactory information, we conducted early gene c-Fos immunostaining in C57BL/6J mice after 10 min of exposure to social odor (male-soiled bedding) (Fig. 1A). Compared to the control group exposed to nonsocial odor (clean bedding), exposure to social odor significantly increases the expression of c-Fos in the MeApv (Fig. 1, B to D). However, no significant increase in c-Fos expression was observed in the MeApd (fig. S1). To identify odor-responsive neuronal populations in the MeApv, we performed c-Fos immunostaining combined with multiplex RNAscope analysis for VGluT2 and vesicular γ-aminobutyric acid transporter (VGAT) after social odor exposure. Quantitative analysis revealed that 75.1% of c-Fos positive neurons expressed VGluT2, whereas only 17.6% exhibited VGAT coexpression (Fig. 1, E and F).

To investigate VGluT2-specific neural encoding of social cues, we transduced Cre-dependent viral construct coding for the calcium-activated green fluorescent protein GCaMP6m [adeno-associated virus (AAV)-EF1α-DIO-GCaMP6m] in the MeApv and implanted optic fibers (Fig. 1, G and H) to record Ca^2+^-associated fluorescence in VGluT2-Cre mice. These recordings were made while the mice encountered either a social cue (male bedding or male mice) or nonsocial cue (clean bedding) (Fig. 1G, right). We observed a significant elevation in calcium signals when mice explored a social odor or a live conspecific but not a control odor (Fig. 1, I to M). Specifically, both the area under the curve (AUC) per second and peak values were significantly greater than the baseline during social exploration (Fig. 1, L to O). When exploring female social odor or a female conspecific, the calcium signals of MeApv^VGluT2^ cells also significantly increased (fig. S2). These results indicate that the MeApv is involved in processing social olfactory information.

### Optogenetic silencing of MeApv VGluT2 neurons induced social recognition impairment

**Fig. 2.**
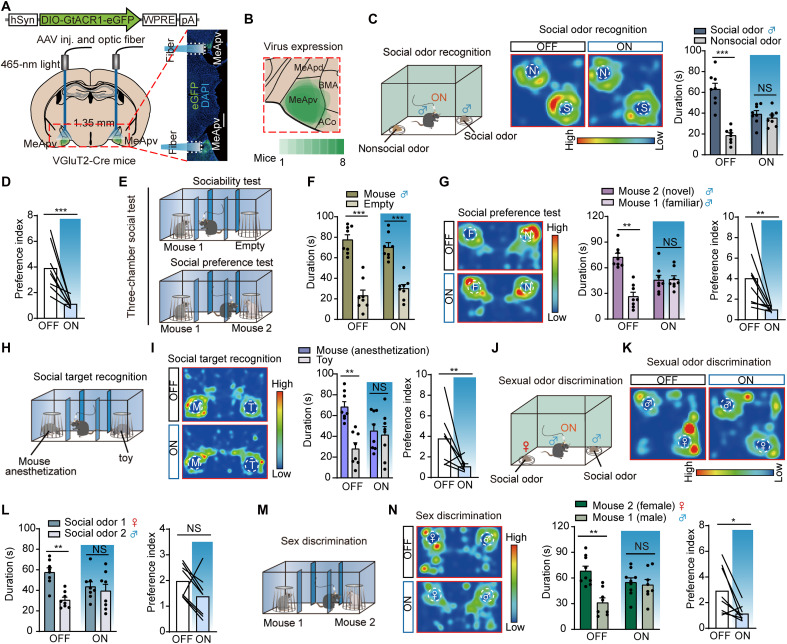
Optogenetic silencing of MeApv VGluT2 neurons impairs social discrimination. (**A**) Virus injection and fiber in VGluT2-Cre mice. Scale bar, 500 μm. (**B**) Virus expression range. (**C**) Left: Social odor recognition test (nonsocial vs. social odor). Middle: Heatmaps. N, nonsocial odor (clean bedding); S, social odor (male bedding). Right: Interaction time (*n* = 8; OFF: *P* < 0.0001; ON: *P* = 0.3590; paired *t* test). (**D**) Social odor preference, OFF versus ON (*n* = 8; *P* = 0.0008, unpaired *t* test). (**E**) Three-chamber social test. (**F**) Sociability test: Time in mouse versus empty chamber (*n* = 8; OFF: *P* = 0.0005; ON: *P* = 0.0008, paired *t* test). (**G**) Left: Heatmaps (F, familiar; N, novel). Middle: Interaction time (*n* = 8; OFF: *P* = 0.0011; ON: *P* = 0.8839; paired *t* test). Right: Social preference, OFF versus ON (*n* = 8; *P* = 0.0082, unpaired *t* test). (**H**) Social target recognition test (anesthetized mouse versus toy). (**I**) Left: Heatmaps (M, mouse; T, toy). Middle: Interaction time (*n* = 8; OFF: *P* = 0.0012; ON: *P* = 0.7085; paired *t* test). Right: Preference, OFF versus ON (*n* = 8; *P* = 0.0093, Mann-Whitney test). (**J**) Sexual odor recognition test (female bedding versus male bedding). (**K**) Heatmaps (♀, female bedding; ♂, male bedding). (**L**) Left: Interaction time (*n* = 8; OFF: *P* = 0.0013; ON: *P* = 0.6492; paired *t* test). Right: Sexual odor preference, OFF versus ON (*n* = 8; *P* = 0.4418, Mann-Whitney test). (**M**) Sex discrimination test (female mouse versus male mouse). (**N**) Left: Heatmaps (♀, female mouse; ♂, male mouse). Middle: Interaction time (*n* = 8; OFF: *P* = 0.0075; ON: *P* = 0.9281; paired *t* test). Right: Preference, OFF versus ON (*n* = 8; *P* = 0.0233, unpaired *t* test). *P < 0.05; **P < 0.01; ***P < 0.001; NS, not significant. Error bar, SEM.

To further establish the role of VGluT2 neurons in encoding social information, we expressed the inhibitory opsin AAV-hSyn-DIO-hGtACR1-enhanced green fluorescent protein (eGFP) into the bilateral MeApv region of VGluT2-Cre mice and positioned optical fibers above the MeApv for selective inhibition of the local neurons (Fig. 2, A and B, and fig. S3). After 3 weeks of recovery, we used an arena (45 cm by 45 cm by 50 cm) under dim light conditions (25 lux) to perform olfactory cue recognition test. We placed clean bedding in one corner and bedding of male mice in the opposite corner of the arena, both covered with a wire containment cup. Normally, mice spend more time investigating odors containing social information (bedding of male mice) when presented with both social and neutral odors (clean bedding). However, when MeApv VGluT2 neurons were inhibited, the mice showed no preference between the social and neutral odors, indicating reduced preference for social odors (Fig. 2, C and D). To determine whether inhibiting MeApv VGluT2 neurons selectively affected social cue processing, we conducted odorant cue recognition experiments. Our results indicated that, without light stimulation, the mice exhibited reduced interest when repeatedly exposed to the same odor, which included responses to both nonsocial and social odors, but significantly increased the exploring time when exposed to a new odor. When MeApv VGluT2 neurons were selectively inhibited, the mice showed a normal decrease in interest toward nonsocial odorants but failed to exhibit similar habituation responses to social odors, suggesting impaired discrimination of social cues while retaining the ability to discriminate nonsocial odorants (fig. S4A). To further verify that inhibiting MeApv VGluT2 neurons does not impair nonsocial odor discrimination, we used tests with more ethologically relevant odors. In the buried food test (fig. S4B), which required fasted mice to locate hidden food using olfactory cues, inhibition of MeApv VGluT2 neurons did not impair olfactory-guided foraging. This was evidenced by similar food-finding latencies in experimental and control groups. Furthermore, this inhibition did not alter freezing behavior in response to the predator odor 2,4,5-trimethyl-3-oxazoline (TMT) (fig. S4C).

Next, we used the classic three-chamber social behavioral paradigm to evaluate socially relevant behaviors. This paradigm includes a sociability test and a social recognition test (Fig. 2E). In the sociability test, control mice with typical social interest spent more time interacting with a compartment containing a stimulus mouse (Fig. 2F) than an empty compartment. In the social recognition test, mice usually spent significantly more time investigating a novel mouse compared to a familiar one. Although inhibition of MeApv VGluT2 neurons did not alter the performance of sociability test, it led to a loss of preference to the novel mice, suggesting impaired social recognition (Fig. 2G). Subsequently, we found that the mice with inhibited MeApv VGluT2 neurons exhibit a typical preference for novel objects in the novel-object recognition test, thereby demonstrating that the deficit in social discrimination was not due to indifference to novelty (fig. S4D).

To verify that olfaction was the dominant sensory modality for social recognition, we designed a modified experiment to reduce most auditory and visual cues and eliminate feedback from the stimulus mouse. In this setup, an anesthetized mouse (enclosed in a wire containment cup) was placed in one chamber, and a toy mouse resembling the anesthetized one in color, shape, and size was placed in the other chamber (Fig. 2H). When MeApv VGluT2 neurons were inhibited, the test mice failed to distinguish between the anesthetized mouse and the toy mouse (Fig. 2I).

We wanted to explore whether MeApv VGluT2 neurons mediated social cue processing of different sexes. Typically, male mice show more interest in female mice and spent more time to explore either the female bedding or female mice when presented together with male bedding or male mice, respectively. However, inhibition of MeApv^VGluT2^ neurons abolished this preference, and the test mice exhibited no bias in exploring male or female mice (Fig. 2, J to N). Light stimulation in eGFP-expressing VGluT2-Cre mice did not alter odor-guided social behavior (fig. S5). These results indicate that inhibition affects recognition of both same-sex and opposite-sex social odors.

Next, we tested the effects of social recognition by optogenetic activation of MeApv^VGluT2^ neurons. We bilaterally expressed AAV-EF1α-DIO-hChR2-eGFP in the MeApv of VGluT2-Cre male mice and implanted an optic fiber above the MeApv for optogenetic stimulation. Neither same-sex nor opposite-sex social discrimination was disrupted by such manipulation (fig. S6).

**Fig. 3.**
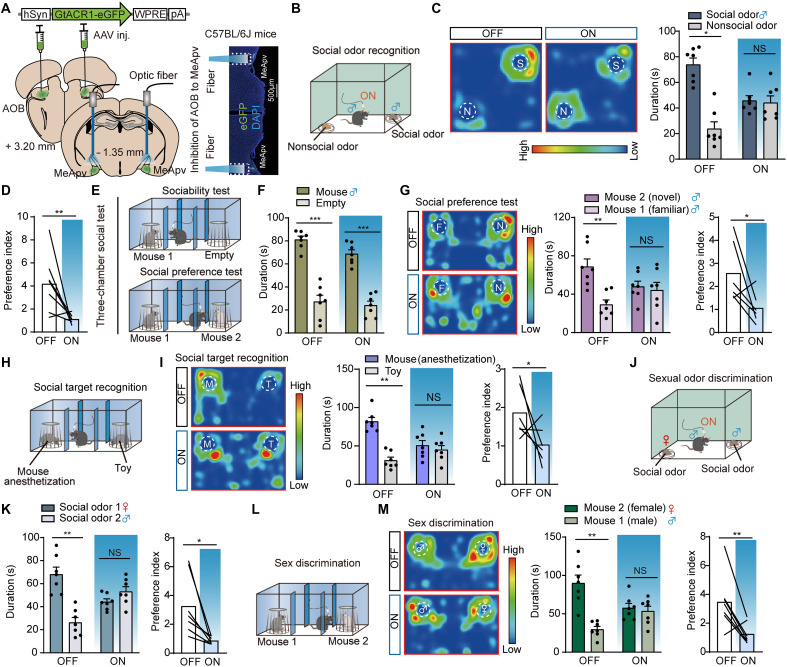
Optogenetic inhibition of the AOB-MeApv pathway induces social discrimination deficits. (**A**) Left: Virus injection and fiber implantation; Right: Representative images of MeApv in GtACR1-expressing mice. Scale bar, 500 μm. (**B**) Social odor recognition test (clean versus male bedding). (**C**) Left: Representative heatmaps. Right: Interaction time (*n* = 7; OFF: *P* = 0.0156, Wilcoxon matched-pairs signed-rank test; ON: *P* = 0.8450, paired *t* test). (**D**) Social odor preference, OFF versus ON (*n* = 7; *P* = 0.0085, unpaired *t* test). (**E**) Three-chamber social test. (**F**) Sociability test: Time in mouse versus empty chamber (*n* = 7; OFF: *P* = 0.0004; ON: *P* = 0.0001, paired *t* test). (**G**) Left: Heatmaps (F, familiar; N, novel mouse). Middle: Novel versus familiar time (*n* = 7; OFF: *P* = 0.0023; ON: *P* = 0.7858; paired *t* test). Right: Social preference, OFF versus ON (*n* = 7; *P* = 0.0167, unpaired *t* test). (**H**) Social target recognition test (anesthetized mouse versus toy). (**I**) Left: Heatmaps (M, mouse; T, toy). Middle: Interaction time (*n* = 7; OFF: *P* = 0.0034; ON: *P* = 0.6355; paired *t* test). Right: Social target preference, OFF versus ON (*n* = 7; *P* = 0.0322, unpaired *t* test). (**J**) Sexual odor discrimination test (female bedding versus male bedding). (**K**) Left: Interaction time (*n* = 7; OFF: *P* = 0.0050; ON: *P* = 0.1607; paired *t* test). Right: Sexual odor preference, OFF versus ON (*n* = 7; *P* = 0.0116, unpaired *t* test). (**L**) Sex discrimination test (female mouse versus male mouse). (**M**) Left: Heatmaps (♀, female mouse; ♂, male mouse). Middle: Interaction time (*n* = 7; OFF: *P* = 0.0014; ON: *P* = 0.7143; paired *t* test). Right: Gender preference, OFF versus ON (*n* = 7; *P* = 0.0023, Mann-Whitney test). *P < 0.05; **P < 0.01; ***P < 0.001; NS, not significant. Error bar, SEM.

Given that previous studies have shown direct projections from the AOB to the MeApv, we investigated whether the AOB-MeApv pathway regulates social information processing. In C57BL/6J male mice, we injected AAV-hSyn-hGtACR1-eGFP bilaterally in the middle-posterior AOB and implanted an optic fiber above the MeApv (Fig. 3, A and B). Inhibition of the AOB-MeApv projection impaired the ability of mice to discriminate social odors (Fig. 3, C and D), distinguish novel from familiar mice (Fig. 3, E to G), differentiate real mice from toy mice (Fig. 3, H and I), and recognize male versus female mice (Fig. 3, J to M).

The MeApv contains two distinct subpopulations of glutamatergic neurons: those expressing VGluT2 and those expressing VGluT1 (*17*). To determine the role of VGluT1 neurons in social recognition, we expressed AAV-hSyn-DIO-hGtACR1-eGFP to the bilateral MeApv in VGluT1-Cre male mice to selectively inhibit VGluT1^+^ neurons and found no significant impact on social olfactory information processing (fig. S7). This indicates that VGluT2 neurons, but not VGluT1 neurons, are critical for modulating social olfactory information.

### Optogenetic activation of MeApv Gad2 neurons induced social recognition impairment

Given the well-established role of balanced excitation and inhibition in precise neural computation and behavior, we hypothesized that disrupting this balance in the MeApv would impair social recognition. We then tested the behavioral impact of manipulating MeApv GABAergic neurons. We expressed AAV-EF1α-DIO-hChR2-eGFP to the left-lateral MeApv in Gad2-Cre male mice and positioned optical fibers above the MeApv region for selective activation the local neurons (fig. S8, A and B). Upon activation, mice exhibited an inability to recognize social odors (fig. S8, C to E), failed to discriminate between novel and familiar mice (fig. S8, F to H), and showed no preference for exploring either male or female mice (fig. S8, I to L). Furthermore, light stimulation of the control cohort (Gad2-Cre mice expressing eGFP) did not affect odor-guided social behaviors (fig. S9). These results indicate that activation of GABAergic neurons disrupts social olfactory recognition, mirroring the deficits observed when VGluT2 neurons were inhibited.

We next explored whether MeApv^VGluT2^ neurons received monosynaptic inputs from MeApv^GABAergic^ neurons. On the basis of the strong colocalization of calcium- and calmodulin-dependent protein kinase IIα (CaMKIIα) and VGluT2 in MeApv neurons demonstrated by our viral labeling (fig. S10), we expressed AAV-hSyn-DIO-ChR2-eGFP and AAV-mCaMKIIα-mCherry (to label VgluT2^+^ cells) into the left-lateral MeApv in Gad2-Cre mice. Using patch-clamp recordings from acute brain slices, we found that four of six (66.7%) recorded mCherry-positive neurons in the MeApv displayed light-evoked inhibitory postsynaptic current in the presence of tetrodotoxin and 4-aminopyridine. The light-induced response could be blocked by pertussis toxin, suggesting that MeApv excitatory neurons receive direct projections from GABAergic neurons (fig. S8B). When we express AAV-hSyn-DIO-hGtACR1-eGFP to the bilateral MeApv for inhibiting GABAergic neurons, there was no effect on social odor recognition (fig. S11).

### Social odor information is retained in the specific VGluT2 cell population

**Fig. 4.**
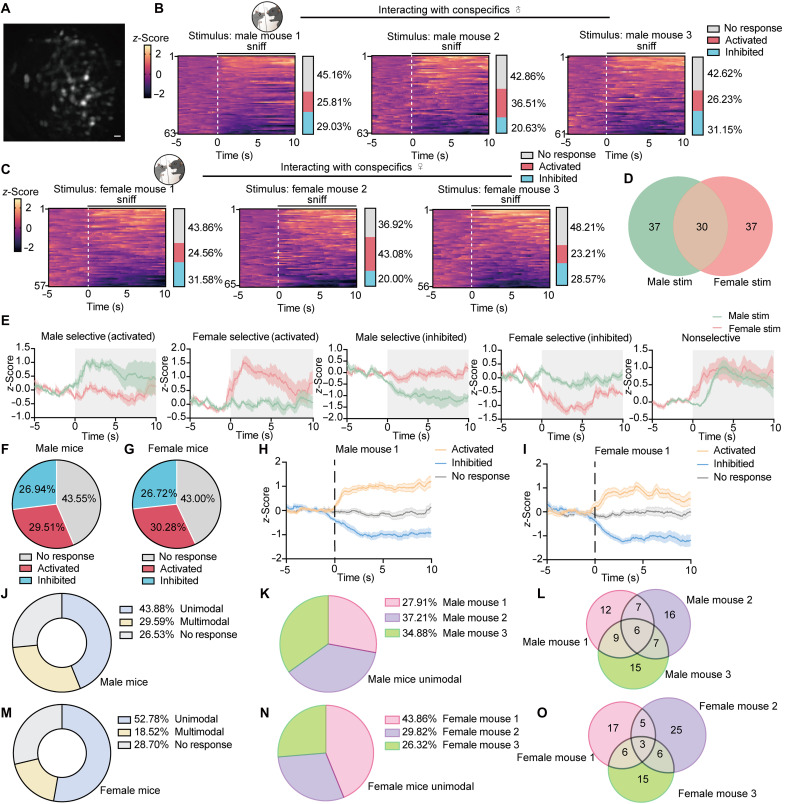
Population encoding of individual male or female conspecifics by MeApv^VGluT2^ neurons. (**A**) Representative raw Ca^2+^ imaging frames acquired through an implanted GRIN lens; scale bar, 20 μm. (**B** and **C**) Left: Heatmap of all recorded neurons showing responses to male (B) or female (C) mouse stimuli (#1 to #3), arranged in order of the AUC during the 0- to 5-s stimulation window (*n* = 3 mice). Right: Proportion of neurons activated, inhibited, or nonresponsive; dashed line, sniff onset. (**D**) Venn diagram showing neurons responding exclusively to male, female, or both; nonresponsive neurons not shown. Numbers represent the total cell counts pooled across all recorded mice. (**E**) Representative single-neuron *z*-scored Δ*F*/*F* traces (*n* = 5 neurons from three mice) aligned to odor onset (time 0). Shaded regions indicate odor presentation. Examples include male-selective activated neurons, female-selective activated neurons, male-selective inhibited neurons, and nonselective neurons responding to both odors corresponding to the response categories quantified in (D). (**F** and **G**) Pie charts showing the average fraction of neurons that were activated, were inhibited, or showed no response in male (F) and female (G) mice. For each sex, proportions were first calculated for each individual mouse (#1 to #3) and then averaged across the three mice to generate the summary charts. (**H** and **I**) Averaged *z*-scored Ca^2+^ signal aligned to sniff onset for male mouse #1 or female mouse #1 stimuli (*n* = 3 mice). Mean, solid line; shaded area, ± SEM. (**J** and **M**) Proportion of neurons classified as unimodal, multimodal, or nonresponsive. (**K** and **N**) Proportion of activated neurons responding to each single stimulus among unimodal neurons. (**L** and **O**) Venn diagrams of the number of neurons responding to individual male (L) or female (O) mouse stimuli.

Next, we explored whether specific MeApv neuronal populations encode social information. By using Cre-dependent AAV vectors to target GCaMP6s expression to VGluT2 neurons, we conducted microendoscopic calcium imaging while presenting social stimuli of three male (same-sex) or three female (opposite-sex) novel conspecifics (Fig. 4). In the behavioral setup, head-fixed subject mice maintained free limb mobility on a rotating platform. Each subject mouse received five 10-s exposures per stimulus mouse (60-s intertrial intervals), with identical parameters for all three stimulus conspecifics. Neuronal activity was continuously recorded throughout the social interactions (Fig. 4A).

Neuronal responses to a given stimulus mouse fell into three categories: activated, inhibited, or nonresponsive (Fig. 4, B to I). The percentiles of neuronal populations that fit in three categories in response to three same-sex or three opposite-sex stimuli were comparable (Fig. 4, B and C). Distribution analysis revealed that separate neuronal populations respond to male, female, or both stimuli (Fig. 4D), which was further illustrated by representative single-neuron traces showing selective activation or inhibition to male or female odors, as well as nonselective responses (Fig. 4E). At the population level, these response patterns were highly consistent across animals. The relative proportions of activated, inhibited, and nonresponsive neurons were largely comparable between individual mice for both male and female stimuli, indicating a stable response composition within MeApv VGluT2^+^ neurons. In addition, alignment of Ca^2+^ signals to sniff onset revealed similar temporal dynamics of stimulus-evoked activity across mice, suggesting reproducible population responses to conspecific odors (Fig. 4, F to I). Furthermore, we found that when test mice were exposed to different male stimulus mice, 43.88% of recorded neurons responded to only a single stimulus mouse, including both activation and inhibition (defined as unimodal); 29.59% responded to multiple stimulus mice (defined as multimodal); and 26.53% showed no response (Fig. 4J). Among all unimodal neurons, 27.91% responded to mouse 1, 37.21% to mouse 2, and 34.88% to mouse 3 (Fig. 4K). Similarly, for female stimuli, 52.78% of labeled neurons were unimodal, while a minority (18.52%) showed multimodal responses (Fig. 4M). Among these unimodal neurons, 29.82% responded to mouse 1, 43.86% to mouse 2, and 26.32% to mouse 3 (Fig. 4N). Population analysis revealed limited response overlap between different stimulus individuals of either sex (Fig. 4, L and O).

**Fig. 5.**
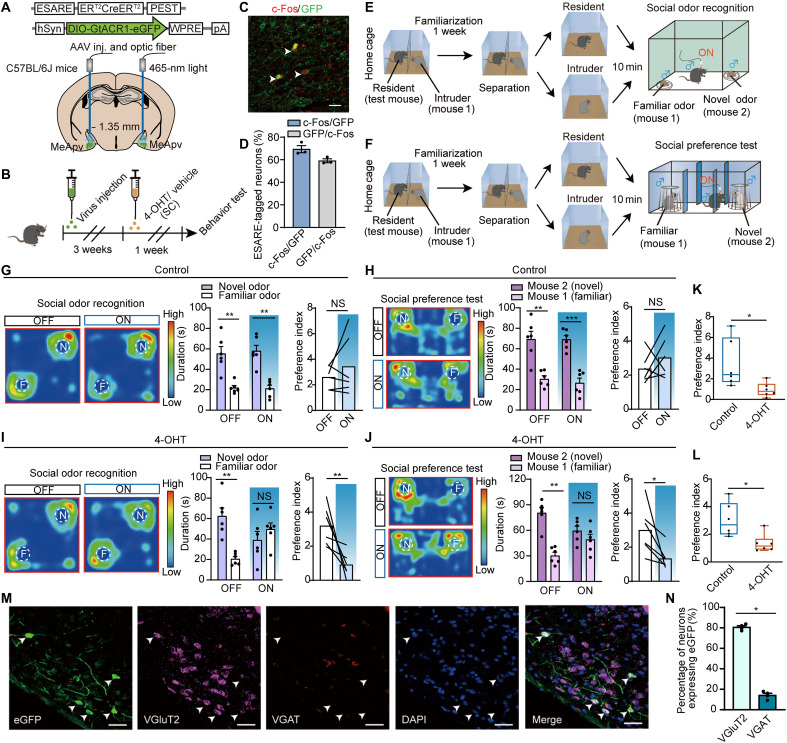
Specific VGluT2 neurons encode and retain social odor memory. (**A**) Virus injection and fiber implantation. (**B**) Experimental strategy. Control mice received identical treatments but no social stimulation. (**C**) ESARE GFP expressing and c-Fos colocalized. Scale bar, 30 μm. (**D**) Overlap of c-Fos–positive and GFP cells. (**E**) Social odor recognition test. (**F**) Social preference test. (**G**) Social odor recognition. Left: Heatmaps [F, familiar odor (mouse 1); N, novel odor (mouse 2)]. Middle: Interaction time (*n* = 6; OFF: *P* = 0.0050; ON: *P* = 0.0074; paired *t* test). Right: Preference, OFF versus ON (*n* = 6; *P* = 0.3717, unpaired *t* test). (**H**) Social discrimination test. Left: Heatmaps (F, familiar mouse; N, novel mouse). Middle: Interaction time (*n* = 6; OFF: *P* = 0.0003; ON: *P* < 0.0001; paired *t* test). Right: Preference, OFF versus ON (*n* = 6; *P* = 0.3138, unpaired *t* test). (**I**) Social odor recognition. Left: Heatmaps (F, familiar odor; N, novel odor). Middle: Interaction time (*n* = 6; OFF: *P* = 0.0028; ON: *P* = 0.4507; paired *t* test). Right: Preference, OFF versus ON groups (*n* = 6; *P* = 0.0022, unpaired *t* test). (**J**) Social discrimination. Left: Heatmaps (F, familiar mouse; N, novel mouse). Middle: Interaction time (*n* = 6; OFF: *P* = 0.0026; ON: *P* = 0.2626, paired *t* test). Right: Preference, OFF versus ON (*n* = 6; *P* = 0.0260, Mann-Whitney test). (**K**) Social odor preference: Control versus 4-OHT (*n* = 6; *P* = 0.0301, unpaired *t* test). (**L**) Social preference: Control versus 4-OHT (*n* = 6; *P* = 0.0152, Mann-Whitney test). (**M**) RNAscope: VGluT2 colocalization in labeled neurons. Scale bars, 50 μm. (**N**) eGFP^+^ cell quantification (*n* = 4 mice; *P* = 0.0286, Mann-Whitney test). *P < 0.05; **P < 0.01; NS, not significant. Error bar, SEM.

We next wanted to explore whether manipulating these conspecific identity–encoding cells could regulate odor-dependent social behaviors. We first expressed an AAV vector expressing CreER^T2^ driven by enhanced synaptic activity–responsive element (AAV-ESARE-CreER^T2^) and AAV-hSyn-DIO-hGtACR1-eGFP in the MeApv of the test mice (*18*). The test mice were then administered 4-hydroxytamoxifen (4-OHT; 40 mg/kg), or corn oil as control, to induce Cre-dependent labeling of neurons related to the following 1-week cohousing with a stimulus mouse (mouse 1) in a perforated barrier-divided cage, allowing olfactory/visual interaction without physical contact (Fig. 5, A and B).

To validate the specificity and efficiency of our approach, we performed dual labeling using the ESARE system (eGFP reporter) combined with c-Fos immunohistochemistry after stimulus mouse exposure. Quantitative analysis showed that 69.36% of ESARE-driven eGFP-positive neurons expressed c-Fos, while 58.17% of c-Fos–positive neurons expressed eGFP (Fig. 5, C and D). Control experiments with 4-OHT administration in the absence of odor stimulation demonstrated minimal background labeling in the MeApv (sparse eGFP^+^/c-Fos^+^ cells; fig. S12). These results collectively validate the system’s specificity and efficiency for tagging activated neuronal ensembles.

Next, we investigated whether selective modulation of social cue–activated neurons would affect animal behaviors. Unlike the control group, optogenetic silencing of these labeled MeApv ensembles impaired both social odor preference (Fig. 5, E and G to K) and social recognition of mouse 1 (Fig. 5, F and I to L). RNAscope staining further confirmed that these labeled neurons were predominantly VGluT2-positive (82.2%) versus VGAT-positive (13.1%) (Fig. 5, M and N).

**Fig. 6.**
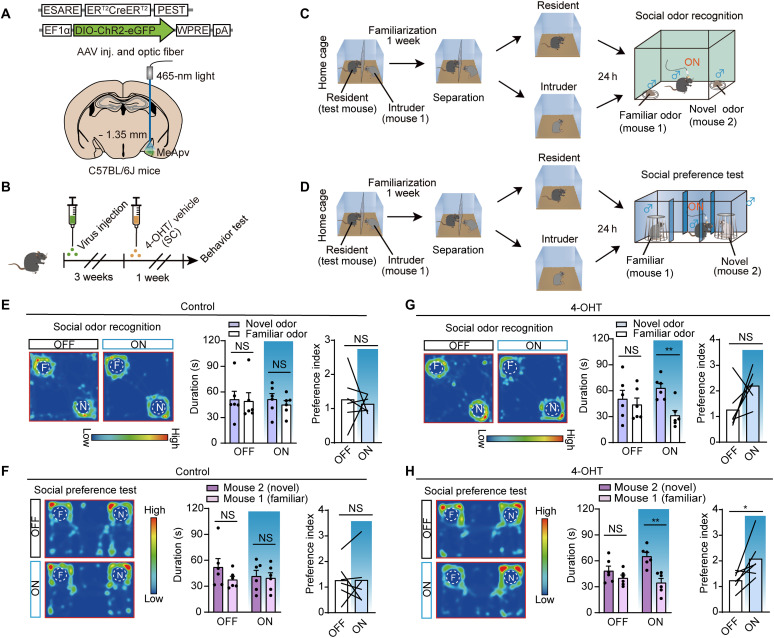
Activation of labeled VGluT2 neurons in the MeApv restores social recognition of familiar conspecifics after 24-hour isolation. (**A**) Schematic of virus injection and fiber implantation. (**B**) Experimental timeline. (**C**) Social odor recognition test design. h, hours. (**D**) Social preference test design. (**E**) Social odor recognition test for the control group. Left: Representative heatmaps. Middle: Time spent with familiar odor versus novel odor (*n* = 6; OFF: *P* = 0.5625, Wilcoxon matched-pairs signed-rank test; ON: *P* = 0.2659, paired *t* test). Right: Social odor preference, OFF versus ON groups (*n* = 6; *P* = 0.6890, unpaired *t* test). (**F**) Social recognition test for the control group. Left: Representative heatmaps. Middle: Time spent with familiar mouse versus novel mouse (*n* = 6; OFF: *P* = 0.5625, Wilcoxon matched-pairs signed-rank test; ON: *P* = 0.8478, paired *t* test). Right: Social preference, OFF versus ON groups (*n* = 6; *P* = 0.9901, unpaired *t* test). (**G**) Social odor recognition test for the 4-OHT group. Left: Representative heatmaps. Middle: Time spent with familiar odor versus novel odor (*n* = 6; OFF: *P* = 0.5729; ON: *P* = 0.0025, paired *t* test). Right: Social odor preference, OFF versus ON groups (*n* = 6; *P* = 0.0649, Mann-Whitney test). (**H**) Social recognition test for the 4-OHT group. Left: Representative heatmaps. Middle: Time spent with familiar mouse versus novel mouse (*n* = 6; OFF: *P* = 0.2612; ON: *P* = 0.0012; paired *t* test). Right: Social preference, OFF versus ON groups (*n* = 6; *P* = 0.0417, unpaired *t* test). *P < 0.05; **P < 0.01; NS, not significant. Error bar, SEM.

Previous research has demonstrated that mice retain social memory for ~24 hours (*19*). Consistent with this finding, control mice failed to discriminate between mouse 1 and mouse 2 after 24 hours of separation, even though the test mice were cohoused with mouse 1 for 1 week. However, when we used an activity-dependent labeling strategy—expressing AAV-ESARE-CreER^T2^ and AAV-EF1α-DIO-hChR2-eGFP in the MeApv of test mice, followed by 4-OHT injection and cohousing with mouse 1—we selectively labeled MeApv neurons encoding mouse 1 (Fig. 6, A and B). Optogenetic activation of these neurons restored the ability to recognize both the odor of mouse 1 and mouse 1 itself 24 hours after separation (Fig. 6, C to H). These results indicate that social information associated with a specific individual (mouse 1) is stably maintained within distinct MeApv neuronal populations.

Next, we examined whether manipulation of the neural population encoding mouse 1 affects recognition of other conspecifics. Using the same activity-dependent labeling strategy, we expressed GtACR1 in MeApv neurons encoding mouse 1 in test mice. After familiarization, optogenetic inhibition of these neurons did not impair the test mice’s ability to discriminate between mouse 2 and mouse 3 (a pair of novel stimulus mice) (fig. S13). Similarly, optogenetic activation of mouse 1 neurons did not enhance recognition of mouse 2 or mouse 3 (fig. S14). Furthermore, when we labeled neurons activated by mouse 1 using the ESARE system and then performed c-Fos immunohistochemistry following exposure to mouse 2, we observed limited colocalization. Only 15.83% of eGFP-positive neurons were also c-Fos–positive, and conversely, 11.57% of c-Fos–positive neurons coexpressed eGFP (fig. S12, C and D).

To evaluate the temporal dynamics of social information encoding in MeApv VGluT2 neurons, we performed longitudinal microendoscopic calcium imaging in a 24-hour test-retest paradigm. On day 1, mice were exposed to different same-sex or opposite-sex conspecifics, and the identical stimulus set was reintroduced on day 2 to assess the stability of neuronal responses (fig. S15, A to C). For each individual stimulus (male mouse 1 or female mouse 1), neurons were classified as stably activated, stably inhibited, reversal response, gained, or lost responses, excluding cells that showed no response on either day. At the population level, comparable response patterns emerged for both male and female stimuli. Quantitative analysis showed that, among neurons that exhibited a response on at least 1 day, 6.8 and 6.0% neurons were stably activated by male and female stimuli, respectively (fig. S15, D to G). Examination of the overall population response rate revealed that 21.19% of neurons were activated by male stimuli on day 1 and 18.54% on day 2 (fig. S15H), whereas 22.08 and 15.67% were activated by female stimuli across the two sessions (fig. S15I). Further analysis of response persistence revealed that only a portion of day 1–responsive neurons maintained their functional state on day 2. Specifically, 14.57% of male-responsive neurons remained stably activated and 9.44% remained inhibited, whereas 14.30 and 8.46% of female-responsive neurons retained activation and inhibition, respectively (fig. S15, J and K). Among responsive neurons, some maintained stable activation or inhibition across days, whereas others either gained or lost responsiveness or switched between activation and inhibition. These results suggest that, although behavioral recognition decays within 24 hours, a persistent subpopulation of MeApv VGluT2 neurons maintains social cue responsiveness. Notably, while this preserved neuronal activity alone cannot sustain individual recognition at the behavioral level, selective activation of these neurons restores discrimination capability—demonstrating their causal role in maintaining social information.

We further tested whether MeApv neurons encode social odor information from opposite sex conspecifics. Optogenetic inhibition of neurons encoding female mouse 1 abolished recognition of female mouse 1 (fig. S16) but did not impair discrimination between female mouse 2 and female mouse 3 (fig. S17). RNAscope staining confirmed that most labeled neurons were VGluT2-positive (75.4%), with only a minority expressing VGAT (17.1%) (fig. S16, I and J).

## DISCUSSION

Our study unveils a previously unrecognized neural mechanism by which mice encode and distinguish individual conspecifics’ social information, advancing the understanding of social recognition at a circuit and cellular level. While prior work established the MeA’s general role in processing social olfactory cues (*10*, *12*, *20*–*26*), we uniquely identify the MeApv subregion, specifically its glutamatergic VGluT2-positive neurons, as both necessary and sufficient for translating olfactory inputs into individual-specific social recognition. Notably, we demonstrate that a small population of MeApv VGluT2 neurons selectively encodes individual conspecific olfactory signatures, enabling mice to discriminate not only gender but also distinct identities. This specificity was revealed through microendoscopic calcium imaging at a single-cell resolution and further confirmed by optogenetic silencing experiments, where disrupting these neurons abolished recognition of related individuals, highlighting their role in fine-grained social memory. These findings suggested the MeApv as a dedicated hub for individual-level social information processing, a function previously attributed to broader amygdalar or cortical networks. Our work provides a mechanistic framework for how social memories are formed and retrieved at the level of single conspecifics, offering insights into disorders characterized by social recognition deficits.

One caveat of our study is that, while we demonstrate the importance of MeApv VGluT2 and Gad2 neurons in the social discrimination of individual conspecifics, a more precise molecular characterization of these cells is lacking. Given the heterogeneous composition of neuronal subtypes in this region—which highly expresses several key receptors, including estrogen receptor α, oxytocin receptor, and androgen receptor—a major future direction is to investigate these distinct subtypes and their potential overlap and interactions with the VGluT2/Gad2 circuits we have described (*27*, *28*). It is important to note that Lin’s group (*29*) recently identified two functional subtypes (primarily GABAergic) within the MeA: Dbx1-lineage neurons, which respond broadly to multiple social cues, and Foxp2-expressing neurons, which specifically regulate intermale aggression. This evidence for functional heterogeneity strongly complements our own results and highlights the importance of future molecular profiling.

Previous studies have highlighted functional heterogeneity among the four subregions of the MeA, including the MeAav, MeAad, MeApd, and MeApv, reflecting differences in cell type composition and afferent input patterns (*30*–*35*). Specifically, the MeApd exhibits highly selective connectivity, primarily receiving projections from the rostral (anterior) region of the AOB while maintaining minimal input from the MOB (*13*). Notably, the rostral AOB is innervated by vomeronasal sensory neurons expressing V1-type receptors, which in rodents detect steroid-derived pheromonal cues in urine (*36*). Previous studies have demonstrated that the MeApd specializes in processing reproductively relevant chemosensory stimuli, consistent with its dense population of androgen and estrogen receptors and its established roles in mating and parenting behaviors (*37*–*39*).

In contrast to the MeApd, the MeApv receives convergent inputs from both MOB and the entire AOB and appears to play a crucial role in processing social information directly transmitted from the AOB (*13*). Several lines of evidence support this conclusion. First, our data show that neurons in the MeApv, but not the MeApd, are significantly activated by same-sex social cues (fig. S1). This observation is consistent with a prior study demonstrating that the MeApd in male mice shows minimal activation to male cues (<10% of neurons being responsive) but robust activation to female cues (>80% of neurons being responsive) (*40*). Second, optogenetic inhibition of AOB-MeApv signaling or local VGluT2 neurons in the MeApv impairs social odor–dependent behaviors, such as conspecific identity recognition and social odor discrimination, without affecting non–social odor–dependent behaviors. Consistent with this, a previous study indicates that disrupting AOB-MeApv signaling impairs social memory (*41*). Third, inhibiting AOB-MeApv signaling or VGluT2 neurons in the MeApv disrupts gender discrimination, suggesting that the MeApv processes social cues from both same-sex and opposite-sex individuals and conveys this information to other regions to regulate gender-dependent social behaviors. The MeApd likely serves as a downstream region for gender-related cues, as it is sexually dimorphic and shows activation patterns consistent with gender-specific social processing (*42*–*44*). Last, the endoscopic calcium imaging and functional labeling experiments showed that subpopulation of VGluT2 neurons in the MeApv encodes social cues from both same-sex and opposite-sex individuals, and their optogenetic manipulation was sufficient to modulate the discrimination of gender and individual conspecifics. Collectively, these findings underscore the critical role of the MeApv in both sensory processing and social behavioral outputs. The temporal reorganization of olfactory-based identity codes likely serves an adaptive function for social memory. It may represent a shift from a stable sensory-driven “tag” for initial recognition toward a more integrated “social engram” that incorporates updated experience, internal state, and behavioral relevance. Such dynamic encoding almost certainly involves top-down modulation. Inputs from the bed nucleus of the stria terminalis and hypothalamus—which regulate social-affective states—and from the cortical amygdala—which processes learned chemosensory valence—are compelling candidate circuits for imparting experience-dependent plasticity onto MeApv representations. Future studies manipulating these pathways will be crucial to test these mechanistic hypotheses.

The link between MeApv function and social behavior has substantial implications for understanding disorders characterized by social deficits, such as autism spectrum disorder (ASD). Sensory processing impairments are among the most common symptoms in ASD, affecting up to 90% of individuals (*45*, *46*). While much attention has focused on visual and auditory processing abnormalities, our findings suggest that olfactory processing, particularly within amygdala circuits, may also be critically involved. Dysfunctions in the amygdala, including its role in sensory integration and social behavior, are well documented in ASD. Studies showing reduced neural discrimination of social stimuli and hyperconnectivity between the amygdala and sensory regions in patients with ASD highlight potential parallels with the mechanisms we propose for MeApv function in rodents (*47*–*49*). These observations warrant further exploration of the MeApv as a model for understanding the neural underpinnings of sensory-social deficits in ASD and other neurodevelopmental disorders.

## MATERIALS AND METHODS

### Animals

Naive wild-type male C57BL/6J mice (RRID: IMSR_JAX:000664), male *Gad2-ires-Cre* (JAX no. 019022), and male *VGluT2-ires-cre* (JAX no. 028863) at 2 to 5 months of age were group housed in clear Plexiglas cages with ad libitum access to food and water until surgery. Mice were maintained on a 12-hour light/dark cycle (starting at 07:00) under a constant temperature of 22° to 23°C and humidity of 40 to 60%. The stimulus mice were 8- to 10-week-old C57BL/6J mice and were group housed. Adult male mice were randomly assigned to either control or experimental groups. Before the experimental period, the animals were housed in the chambers within custom-designed stainless-steel cabinets. All experimental procedures were approved by Zhejiang University Animal Experimentation Committee (approval number: AIRB-2021-948).

### Viral injection and stereotaxic surgeries

AAV-EF1α-DIO-GCaMP6m (AAV2/9, 5.0 × 10^12^ genomic copies/ml) and AAV-hSyn-DIO-mCherry (AAV2/9, 5.0 × 10^12^ genomic copies/ml) were made by Brain Case Co., Ltd. (Shenzhen, China), and AAV-hSyn-hGtACR1-eGFP (AAV2/9, 1.0 × 10^13^ genomic copies/ml), AAV-hSyn-DIO-hGtACR1-eGFP (AAV2/9, 1.0 × 10^13^ genomic copies/ml), AAV-hSyn-DIO-eGFP (AAV2/9, 1.0 × 10^13^ genomic copies/ml), AAV-EF1α-DIO-hChR2-eGFP (AAV2/9, 1.0 × 10^13^ genomic copies/ml), and AAV-mCaMKIIα-mCherry (AAV2/9, 1.0 × 10^13^ genomic copies/ml) were made by Shanghai Taitool Bioscience Co., Ltd. (Shanghai, China). AAV-ESARE-ER^T2^CreER^T2^-PEST (AAV9, 1.0 × 10^14^ genomic copies/ml) was made by Vigene Biosciences Co., Ltd. (Shanghai, China). AAV-CAG-Flex-GCaMP6s-WPRE-SV40 (AAV5, 2.2 × 10^13^ genomic copies/ml) was made by Addgene (America). AAV-mCaMKIIα-eGFP (AAV2/9, 5.0 × 10^12^ genomic copies/ml) was made by BrainVTA Co., Ltd. (Wuhan, China).

For optogenetic surgeries, mice were deeply anesthetized with sodium pentobarbital (1% w/v). Anesthetized mice were placed in a stereotactic instrument (RWD Life Science) with the body temperature maintained at 37°C using a heating pad. Then, 50 to 100 nl of virus solution (volume adjusted on the basis of viral titer and expression strength) was injected into the target region at a rate of 20 nl/min. The viral injection sites included the MeApv and the AOB. Stereotaxic injection of AAV-hSyn-DIO-hGtACR1-eGFP or AAV-EF1α-DIO-hChR2-eGFP was given into the bilateral or left-lateral MeApv [anteroposterior (AP), −1.35 mm; mediolateral (ML), ±1.85 mm; dorsoventral (DV), −5.50 mm] in VGluT2-Cre mice or Gad2-Cre mice. Stereotaxic injection of AAV-hSyn-hGtACR1-eGFP was given into the bilateral AOB (AP, 3.20 mm; ML, ±1.00 mm; DV, −2.20 mm) in C57BL/6J mice. After the injection, an implantable optic fiber [200-μm core diameter; 6 mm long; 0.37 numerical aperture (NA); Inper Inc.] was implanted above the MeApv (AP, −1.35 mm; ML, ±1.85 mm; DV, −5.35 mm), and then dental cement was used to secure the optic fiber to the skull.

For in vivo calcium recordings, stereotaxic injection of AAV-EF1α-DIO-GCamp6m was given into the left-lateral MeApv (AP, −1.35 mm; ML, +1.85 mm; DV, −5.50 mm; 150 nl) in VGluT2-Cre mice at a rate of 20 nl/min. After the injection, an implantable optic fiber (200-μm core diameter; 6 mm long; 0.37 NA; Inper Inc.) was implanted above the injection site (MeApv: AP, −1.35 mm; ML, ±1.85 mm; DV, −5.35 mm), and then dental cement was used to secure the optic fiber to the skull.

For functional labeling experiments, stereotaxic injection of AAV-hSyn-DIO-hGtACR1-eGFP or AAV-EF1α-DIO-hChR2-eGFP and AAV-ESARE-ER^T2^CreER^T2^-PEST was given into the bilateral or left-lateral MeApv (AP, −1.35 mm; ML, ±1.85 mm; DV, −5.50 mm) in C57BL/6J mice. After the injection, an implantable optic fiber (200-μm core diameter; 6 mm long; 0.37 NA; Inper Inc.) was implanted above the MeApv (AP, −1.35 mm; ML, ±1.85 mm; DV, −5.35 mm), and then dental cement was used to secure the optic fiber to the skull.

For whole-cell recording experiments, stereotaxic injection of AAV-EF1α-DIO-hChR2-eGFP and AAV-mCaMKIIα-mCherry was given into the left-lateral MeApv (AP, −1.35 mm; ML, +1.85 mm; DV, −5.50 mm) in Gad2-Cre mice. After surgery, mice were returned to their cages and given 3 to 4 weeks for recovery and viral expression before subsequent experiments.

Animals were allowed a minimum 3-week recovery period before behavioral testing and whole-cell voltage-clamp recordings. Following behavioral testing, injection sites and fiber implant placements were confirmed using confocal microscopy on brain tissue sections. Mice with mistargeted injections or implants were excluded from this study.

### In vivo optogenetic stimulation

Mice that underwent virus injection and optical fiber implantation were used for all optical stimulation behavior tests. All test mice were habituated to the fiber connection for at least 2 days before testing. On the experiment day, the cannula was connected via an optic fiber sleeve to a 465-nm laser.

In all (ChR2) behavior tests, the power of the laser was 0.5 to 2 mW with a repetition rate of 10 Hz and a laser pulse width of 5 ms. Laser stimulation was delivered 30 s/min. In all (GtACR) behavior tests, the power of the laser was 6 to 8 mW with continuous current. Any mice with missed injections or cannula locations were excluded.

### In vivo calcium recordings and analysis

GCaMP Fiber Photometry System (Inper) was used (470 nm, 30 to 40 μW). Ca^2+^ signals from VGluT2-Cre male mice were analyzed during their exploration of different stimuli including clean bedding, male bedding, and the presence of other male mice. GCaMP fluorescence was detected by a fiber photometry system (Thinkerbiotech, China). Raw signals were demodulated and analyzed with custom-written software in MATLAB. For the quantification of transient amplitudes and event-triggered average plots, a perievent window was defined from 0 to +5 s around the event of interest (sniffing bedding or another mouse). This window constitutes our region of interest for temporal analysis. The 1-s window was immediately before the onset of an exploration event, which was used to calculate the reference fluorescence (*F*_0_). The 5-s window was after exploration onset, during which Δ*F*/*F*_0_ = (*F* − *F*_0_)/*F*_0_ was computed to normalize neural activity. Exploring events that were at least 5 s long were analyzed. The exploring events were manually scored on the basis of the recorded video by using a customized MATLAB code. Data were discarded when viral expression was not restricted to the MeApv or the optic fiber was not targeting the MeApv.

### Slice preparation

Mice were sedated with sodium pentobarbital and perfused with an ice-cold oxygenated cutting artificial cerebrospinal fluid (ACSF) containing 110 mM choline chloride, 2.5 mM KCl, 1.3 mM NaH_2_PO_4_, 7 mM MgCl_2_·6H_2_O, 0.5 mM CaCl_2_·2H_2_O, 25 mM NaHCO_3_, and 20 mM d-glucose. Then, brains were removed rapidly and submerged in cutting solution for 1 min. Coronal slices (300 μm) were cut on a Leica VT1200S vibratome and then incubated in standard ACSF containing 125 mM NaCl, 2.5 mM KCl, 2 mM CaCl_2_·2H_2_O, 1.3 mM NaH_2_PO_4_, 25 mM NaHCO_3_, 1.3 mM MgCl_2_·6H_2_O, and 10 mM d-glucose at 32°C for 30 min. After that, slices were recovered at room temperature for 1 hour.

### Patch-clamp recording

After recovery, slices are held in a recording chamber. Oxygenated standard ACSF is continuously perfused over the slice. The incoming perfusate was warmed by a feedback-controlled heater to maintain a solution temperature at the slice of 32 ± 1°C.

MeApv neurons expressing mCherry were visualized using an upright microscope with a 40× water immersion objective (Olympus BX51WI).

Whole-cell voltage-clamp recordings were performed with borosilicate pipettes filled with a low-chloride ion concentration intracellular solution containing 140 mM CsCl, 10 mM Hepes, 4 mM MgCl_2_·6H_2_O, 0.5 mM EGTA, 4 mM Na_2_ATP, 0.4 mM Na_4_GTP, and 10 mM QX-314 (pH 7.3, 297 mosm). Electrodes had resistances between 3 and 5 MΩ.

To activate ChR2-expressing axons, brief blue light pulses were delivered onto the recorded cell. Optogenetically induced inhibitory postsynaptic currents were recorded by holding the membrane potential of recorded neurons at −70 mV, while the bath solution contained an AMPA/kainic acid–type glutamate antagonist (10 μM DNQX). ACSF, tetrodotoxin (1 μM), and 4-aminopyridine (100 μM) were sequentially used to test whether optogenetically evoked responses are monosynaptic, followed by the addition of pertussis toxin (50 μM) to block the γ-aminobutyric acid type A receptor. All drugs were preapplied for 5 min in the slice chamber before data acquisition.

Signals were filtered at 2 kHz and digitized at 10 kHz using a MultiClamp 700B amplifier and DigiData1550B (Molecular Devices). Data were analyzed using Clampfit (Molecular Devices). Latency was measured as the time difference when the current exceeded 1.5-fold the standard deviation of the baseline compared to the light onset.

### Behavioral experiments

Adult male mice (aged 2 to 5 months) were used for all behavior tests. Mice were individually habituated to the investigator by being handled several times before their first behavior test. Mice were habituated to transportation from the feeding room to the testing room for 3 days. On the experimental day, mice were acclimated for more than 1 hour after being transported to the testing room. For the stimulation experiments, behavioral test was performed at least 3 to 4 weeks following viral injection to allow sufficient time for recovery from surgeries and transgene expression. All behavioral videos were recorded using the ANY-maze video tracking system (Stoelting Co.). Social preference, social odor preference, social target preference, and sex odor preference were calculated on the basis of the time spent interacting with a novel versus familiar mouse, a social versus nonsocial odor, an anesthetized mouse versus a toy mouse, and female-soiled versus male-soiled bedding, respectively.

#### Three-chamber test

We used a modified procedure on the basis of previous descriptions (*50*). The three-chamber apparatus consisted of a Plexiglas rectangular box divided into three equally sized compartments. In the first stage, a test mouse was placed in the center compartment and allowed to habituate to the apparatus for 10 min. In the second stage (sociability test), a stimulus mouse (age- and sex-matched C57BL/6J novel mouse) was placed in a wire containment cup in the left or right compartment (20 cm by 40 cm by 21 cm). An empty wire containment cup was placed in the other compartment. The test mouse was then allowed to explore the apparatus freely for 10 min. The time spent by the test mouse investigating each containment cup was measured. In the third stage (social preference test), another age- and sex-matched C57BL/6J novel mouse was placed in a wire containment cup on the other side, and the test mouse was then allowed to explore the chambers freely for 10 min. The time spent by the test mouse investigating the cup containing either the familiar (first stimulus mouse) or the stranger (second stimulus mouse) was calculated. The placement of the stimulus mouse on the left or right side of the chamber was systematically alternated between trials to eliminate any chamber bias.

For stimulation experiments, light was permanently applied during the whole sociability or social preference test. For functional labeling experiments, 4-OHT (40 mg/kg) or corn oil as control was injected subcutaneously 3 weeks after viral expression, followed by cohousing with a stimulus mouse in a shared environment, and the mice were separated by a perforated transparent partition for more than 1 week before behavioral tests.

#### Odor recognition test

The odor recognition test was performed to assess olfactory sensation in an open-field arena (45 cm by 45 cm by 50 cm) under dim light conditions (25 lux). The test mice were individually placed in the center of the box and allowed to explore freely for 5 min. Clean bedding was placed in one dish, which was then positioned in one corner of the box. Male/female–soiled bedding was placed in the other dish, which was positioned diagonally opposite to the first dish. The time spent exploring the clean bedding and male/female–soiled bedding was calculated. For the stimulation experiments, optical stimulation was applied during the test phase. The positions of the dishes containing clean or male/female bedding were counterbalanced across animals within a group and between the control and experimental groups. Behavioral videos were recorded using an ANY-maze video tracking system.

#### Testing for identifying anesthetized mice and toy mice

We refined the protocol on the basis of an earlier study (*50*). The discrimination test used the three-chamber apparatus under dim light conditions (25 lux). The test mouse was placed in the middle compartment and allowed to habituate to the apparatus for 10 min. An anesthetized mouse was placed inside a wire containment cup in either the left or right compartment (20 cm by 40 cm by 21 cm). The toy mouse (familiar in shape and color) was placed in the other compartment. The test mouse was then left to explore the apparatus for 10 min. The time spent by the test mouse investigating each containment cup was measured. The placement of the stimulus mouse on the left or right side of the chamber was randomized between trials to eliminate any chamber bias.

#### Sex discrimination test

We adapted the protocol on the basis of an earlier study (*50*).The sex discrimination test used the three-chamber apparatus under dim light conditions (25 lux). The test mouse was placed in the middle compartment and allowed to habituate to the apparatus for 10 min. A stimulus mouse (an age-matched C57BL/6J male) was placed inside a wire containment cup in either the left or right compartment (20 cm by 40 cm by 21 cm). Another stimulus mouse (an age-matched C57BL/6J female) was placed in the other compartment. The test mouse was then left to explore the chambers for 10 min. The time spent by the test mouse investigating each containment cup was recorded. The placement of the stimulus mouse on the left or right side of the chamber was systematically alternated between trials to eliminate any chamber bias.

#### Novel object recognition test

This test was performed under the same environmental conditions as the odor recognition test. The procedure included a training phase and a test phase, separated by a 1-hour interval. During training, the mouse was placed in an arena for 10 min with two identical objects (either two Lego brick towers or two Falcon tissue culture flasks filled with sand, as previously described for object recognition tests). The objects were positioned near the corners of one wall. After the 1-hour interval, the test phase began. The mouse was returned to the arena for 5 min, where one familiar object (the same type as in training) and one novel object (the alternative type) were placed in adjacent corners. The location of the novel object was systematically alternated between trials to control for any potential corner bias.

#### Odor habituation test

This test was as described in the previous literature (*51*). Briefly, the test involves presenting four odors sequentially using cotton swabs: vanilla, banana, social odor 1, and social odor 2. Social odors were collected from male- or female-soiled bedding. Each odor was presented three times, each for 2 min, with a 1-min interval between trials. The time spent by the mice investigating the cotton swabs was recorded using a stopwatch.

#### Buried food test

On day 1, mice were fasted for 24 hours with water available ad libitum. On day 2, two identical cages (40 by 20 cm) were prepared: one for acclimation and one for testing. Both were filled with ~5 cm of clean bedding. A piece of standard chow was buried 5 cm beneath the bedding in one corner of the test cage. Each mouse was first placed in the acclimation cage for 5 min. Subsequently, it was transferred to the test cage and allowed to explore freely for 5 min. The latency to find the food was recorded. The bedding in both cages was replaced between trials to eliminate potential odor cues. Data from mice that did not move during the test were excluded as invalid.

#### Predator odor–induced freezing test

This test was performed under the same environmental conditions as the odor recognition test, a 3-cm petri dish with a lid was fixed in one corner of the open field by double-sided tape, and the petri dish was placed with a drop of filter paper with innate fear odor—a complex of similar components in fox dung, TMT. During the testing phase, mice were placed into the open field, the lid of the petri dish was removed, the mice were free to move around the field for 5 min before being taken out, and at the same time, the lid of the petri dish was put back on. After each mouse finished the test, the behavioral device was swabbed twice with 75% alcohol, then the residual alcohol was dried with a dry paper towel, and after the alcohol completely evaporated, the next mouse was placed in the behavioral test. The behavioral room was kept ventilated throughout to avoid excessive levels of TMT in the environment.

### RNAscope

Mice were deeply anesthetized with sodium pentobarbital and then euthanized. Intracardial perfusion was then conducted with 0.9% saline followed by 4% paraformaldehyde in 0.1 M phosphate-buffered saline (PBS). The brain was removed and placed in 4% paraformaldehyde at 4°C for 6 to 8 hours and then transferred to 30% sucrose solution for at least 36 hours at 4°C. Coronal brain sections were cut using a freezing microtome (Leica) at 16 μm. RNAscope Multiplex Fluorescent Detection Kit v2 [Advanced Cell Diagnostics Inc. (ACD), 323110] was used to label activated neurons with probes indicated in figures following the User Manual of ACD (Document 323100-USM). Customized fluorescent RNAscope probes for *Slc32a1* (VGAT; ACD, 319191-C2) and *Slc17a6* (VGluT2; ACD, 319171) were also ordered from ACD. Images were acquired with the Olympus FV3000 and processed with ImageJ software (National Institutes of Health).

### Immunohistochemistry and imaging

Mice were deeply anesthetized with sodium pentobarbital and then euthanized. Intracardial perfusion was then conducted with 0.9% saline followed by 4% paraformaldehyde in 0.1 M PBS. The brain was removed and placed in 4% paraformaldehyde at 4°C for 6 to 8 hours and then transferred to 30% sucrose solution for at least 36 hours at 4°C. Coronal brain sections were cut using a freezing microtome (Leica) at 30 or 40 μm. For staining, sections were washed three times in 0.01 M PBS, rinsed with 0.3% Triton X-100 in 0.1 M PBS (30 min) or frozen methanol (10 min at −20°C), and then blocked with 10% normal bovine serum for 1 hour at room temperature. Sections were incubated with the primary antibody anti–c-Fos (1:800, gp, Sysy) overnight at 4°C. After rinsing, sections were incubated with a fluorophore-conjugated secondary antibody (gp Cy3 for Figs. 1 and 4 and fig. S8 and gp Cy5 for fig. S1) for 1 hour at room temperature (1:1000; Jackson).

For Fig. 4 (C and D) and fig. S8 (I and J), we used C57BL/6J mice injected with AAV-ESARE-ER^T2^CreER^T2^-PEST and AAV-hSyn-DIO-eGFP into the MeApv. After 3 weeks for viral expression, mice received a subcutaneous injection of 4-OHT. Thirty minutes later, they were exposed to a novel mouse (mouse 1) for 10 min. For Fig. 4 (C and D), mice were perfused 90 min after socialization, and brains were stained for c-Fos. In this case, the ESARE system and c-Fos staining both report on neural activity from the interaction with mouse 1. For fig. S8 (I and J), 1 week later, the same mice were exposed to a second novel mouse (mouse 2) for 10 min and perfused 90 min afterward for c-Fos staining. Here, the ESARE system labels neurons activated by mouse 1, while c-Fos identifies neurons activated by mouse 2.

The nuclei were stained with DAPI (4′,6-diamidino-2-phenylindole), and the slices were mounted on microscope slides. Images were captured using an Olympus FV-1000 (×10) or Olympus FV-3000 (×20) inverted confocal microscope. For quantification of c-Fos–positive cells, 12 sections from four mice or 9 sections from three mice (three sections per mouse) were used. Cell counts and analysis were performed using ImageJ software (National Institutes of Health).

### Microendoscopic calcium imaging and data analysis

For in vivo microendoscopic imaging, AAV-CAG-Flex-GCaMP6s was injected into the MeApv of VGluT2-Cre mice (200 nl). Three weeks after viral injection, a 500-μm-diameter, 7-mm-length gradient-index (GRIN) lens was implanted 0.1 to 0.2 mm above the target region (Inper Co., Ltd). One week after base implantation, a custom miniature microscope was gradually lowered above the GRIN lens. When GCaMP-expressing neurons became clearly visible under the microscope, the baseplate was fixed with dental cement. Before the behavioral test, mice were habituated to wearing the miniscope at least three times within 1 week. Calcium fluorescence was recorded at 20 frames/s using InperSight software. To minimize motion-related artifacts, calcium imaging was performed in head-fixed mice with the body allowed to move freely. For each mouse, one video was acquired per stimulus mouse each day. For the assessment of sex-dependent responses, mice were placed on a clean rotating platform. Recording sessions consisted of a 60-s baseline and 10-s access to stimulus mice from the first attachment, followed by a 60-s interval between recordings. During each session, mice were allowed to make at least five repeated trials with the stimulus mouse, each lasting longer than 10 s. All behaviors were recorded with a synchronized camera (Logitech) during calcium imaging.

Raw videos were processed by using InperSight Analysis software (Inper Inc. based on CNMF-E) for deglowing, denoising, background correction, and motion correction. Cells exhibiting abnormal fitted calcium signals were excluded. Ca^2+^ fluorescence signals were *z*-score normalized relative to the prestimulus baseline. Δ*F*/*F* was calculated as (*F* – *F*_0_)/*F*_0_, where *F*_0_ denotes the mean baseline fluorescence. Each cell was assigned a unique identifier for each experimental day to enable cross-session registration and alignment of neuronal activity across recordings. To ensure comparability between first-day analyses of male and female stimulus mice and across-day comparisons, videos were realigned during processing. Spontaneous neuronal activity was assessed during undisturbed 10-min periods preceding each stimulation session, and neuronal activity was quantified as the AUC of the Δ*F*/*F* trace.

Neurons were classified as “activated,” “inhibited,” or “nonresponsive” on the basis of *z*-score thresholds. Neurons were classified as activated when their *z*-score exceeded 1 for at least four consecutive frames during the stimulus period, inhibited if their *z*-score decreased below −1 for at least four consecutive frames, and nonresponsive if neither criterion was satisfied. Stimulus selective neurons were quantified by evaluating each neuron’s responses to individual stimulus mice, and the proportion of neurons responsive to each specific stimulus mouse was calculated as the number of neurons exhibiting significant responses to that stimulus mouse divided by the total number of recorded neurons. For neurons recorded across multiple days, their responses to the same stimulus mouse on different experimental days were analyzed to assess response stability. Neurons were considered stable if they maintained the same response type across days, lost if they were responsive on the first day but became nonresponsive, or gained if they were nonresponsive on the first day but became responsive or exhibited a reversal response if their response type switched from activation to inhibition or vice versa. Cross-day response overlap was quantified for each stimulus mouse as the proportion of neurons that maintained the same response type across consecutive days. Analyses were performed independently for each subject mouse and corresponding stimulus mouse, and results were subsequently pooled across the population.

### Statistics

Sample size was referred to previous studies in the same field. In all the experiments, data acquisition and analysis were performed in a double-blinded manner. All the results are presented as the means ± SEM. All statistical analyses were performed using GraphPad Prism software. All data were first tested for normality before statistical testing. For within-subject comparisons (e.g., the same mouse exposed to familiar versus stranger stimuli), paired *t* tests (normally distributed data) or Wilcoxon signed-rank tests (nonnormal distributions) were applied. Between-group comparisons used unpaired *t* tests or Mann-Whitney *U* tests depending on distribution. Parametric tests were restricted to normally distributed data (*n* ≥ 6), while nonparametric tests were performed for small samples (*n* < 6) or skewed distributions. The significance level was set as *P* < 0. 05 (*P < 0. 05, **P < 0. 01, and ***P < 0. 001).
