# Supplementary material for: The posteroventral part of the medial amygdala nucleus glutamatergic neurons encodes conspecifics’ individual identity in rodents
Source: Sci Adv. 2026 May 22;12(21):eady9830. doi: 10.1126/sciadv.ady9830 (PMC13196748; doi:10.1126/sciadv.ady9830)
Supplement: Supplementary file 1 — Figs. S1 to S17 [file sciadv.ady9830_sm.pdf]

Science Advances
AAAS

Supplementary Materials for

The posteroventral part of the medial amygdala nucleus glutamatergic neurons encodes conspecifics' individual identity in rodents

Lu Zheng *et al.*

Corresponding author: Libiao Pan, libiao_pan@zju.edu.cn; Hongbin Yang, hongbinyang@zju.edu.cn; Gao Chen, d-chengao@zju.edu.cn; Hao Wang, haowang@zju.edu.cn

Sci. Adv. **12**, eady9830 (2026)
DOI: 10.1126/sciadv.ady9830

This PDF file includes:

Figs. S1 to S17

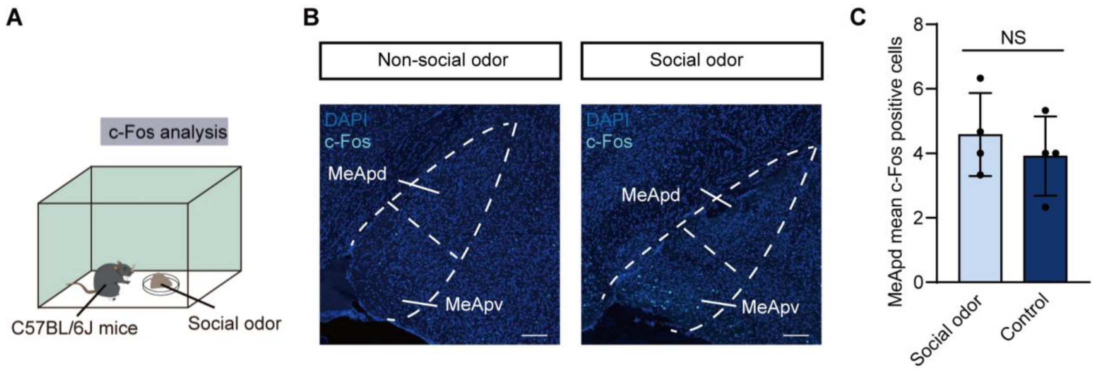

Fig. S1. c-Fos expression in the MeApd in response to social olfactory cues (related to Figure 1).

(**A**) Schematic of c-Fos analysis following exposure to male bedding.

(**B**) Representative images of c-Fos immunoreactivity in the MeApd. Scale bars, 100 μm.

(**C**) Quantification of c-Fos-positive cells in the MeApd (n = 4 mice per group, P = 0.6857, Mann-Whitney test).

NS, not significant. Error bar, SEM.

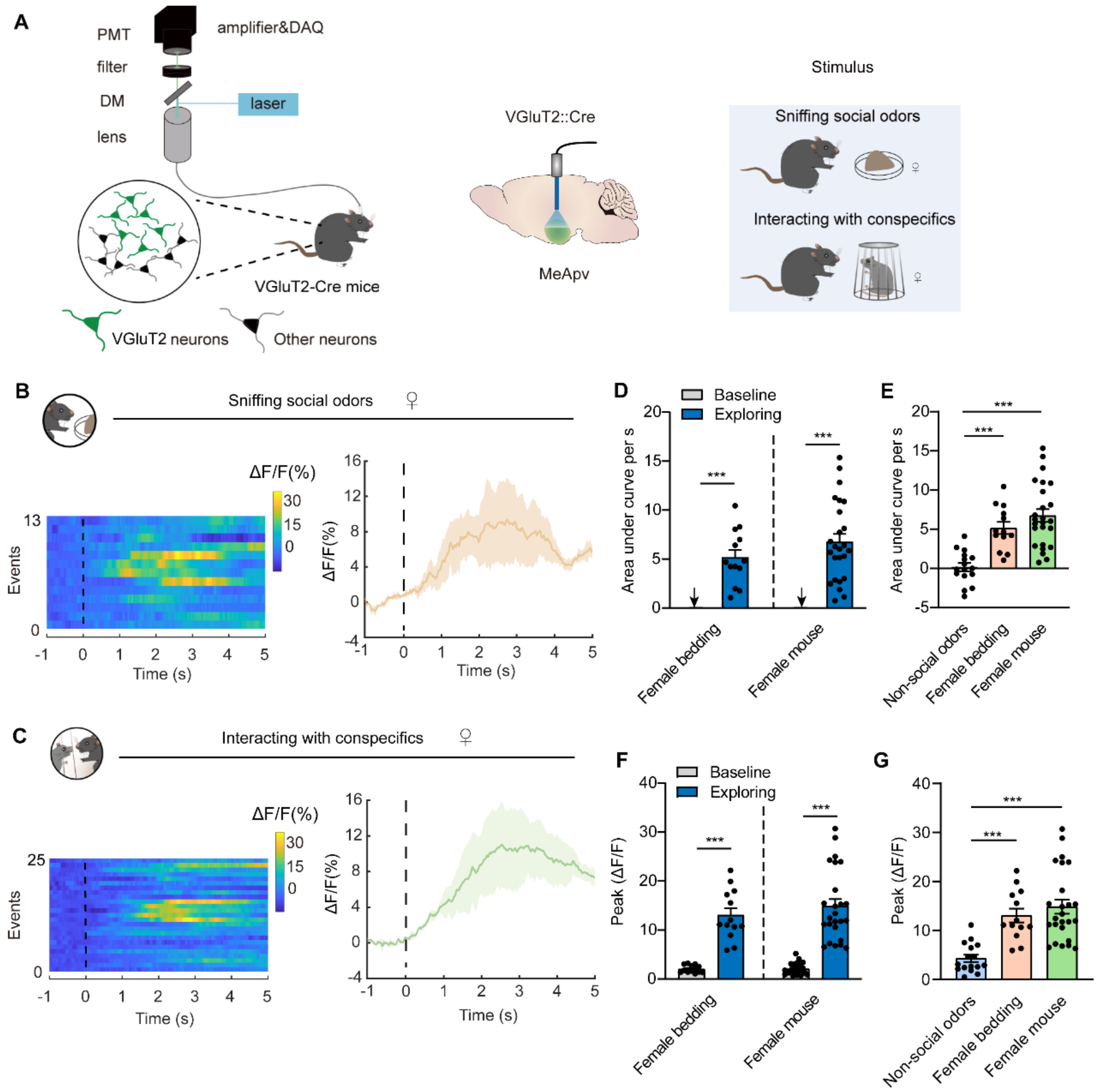

Fig. S2. MeApv VGluT2 neurons exhibit high activity in response to social cues from opposite-sex conspecifics (related to Figure 1).

(**A**) Schematic of calcium signal recording.

(**B**) Heatmap and averaged GCaMP6m signals during sniffing of female social odors.

(**C**) Heatmap and averaged GCaMP6m signals during interaction with conspecifics.

(**D, E**) Area under the curve (AUC) per s of GCaMP6m signals in response to female social and non-social cues. (D) For female social odors, n = 13 events from 3 mice, $P <$ 0.0001; for female mice, n = 25 events from 3 mice, $P < 0.0001$; unpaired t-test. (E) Non-social odors *vs.* social odors, $P < 0.0001$, non-social odors *vs.* female mice, $P <$ 0.0001; n = 16 events from 3 mice for non-social odors, unpaired t-test.

(**F, G**) Peak amplitude of GCaMP6m signals in response to female social and non-social cues. (F) For female social odors, n = 13 events from 3 mice, $P < 0.0001$; for female

mice, *n* = 25 events from 3 mice, *P* < 0.0001; Mann-Whitney test. (G) Non-social odors *vs.* social odors, P < 0.0001, non-social odors *vs.* female mice, P < 0.0001; Mann-Whitney test.

*** P < 0.001. Error bar, SEM.

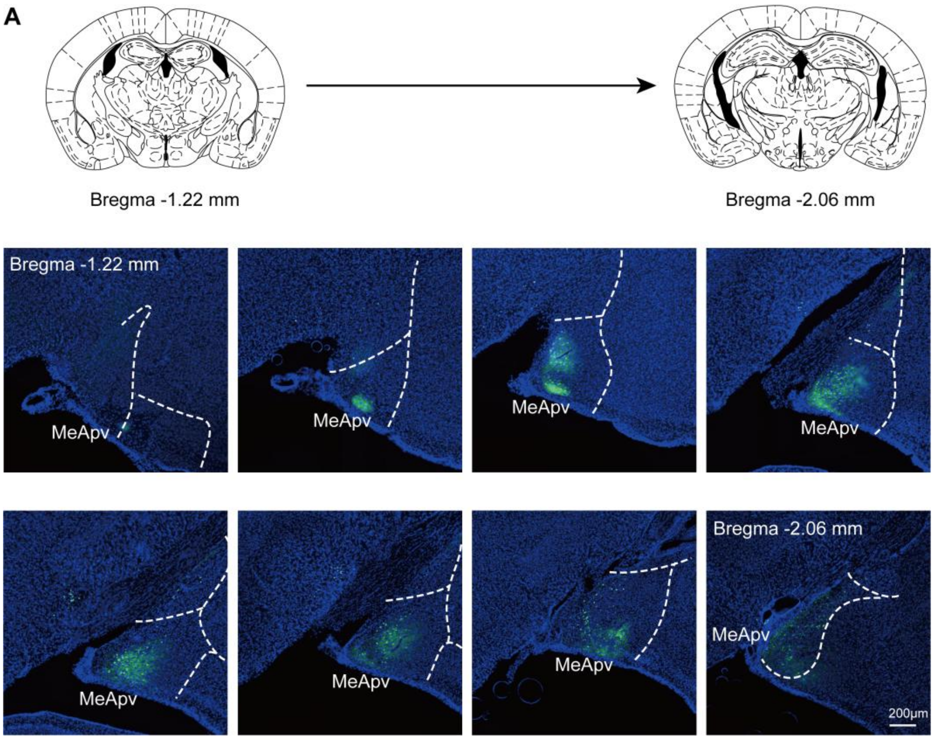

Fig. S3. Viral vector injection site in the MeApv (related to Figure 2).

(**A**) Representative image showing viral vector expression in the MeApv. Scale bar, 200 μm.

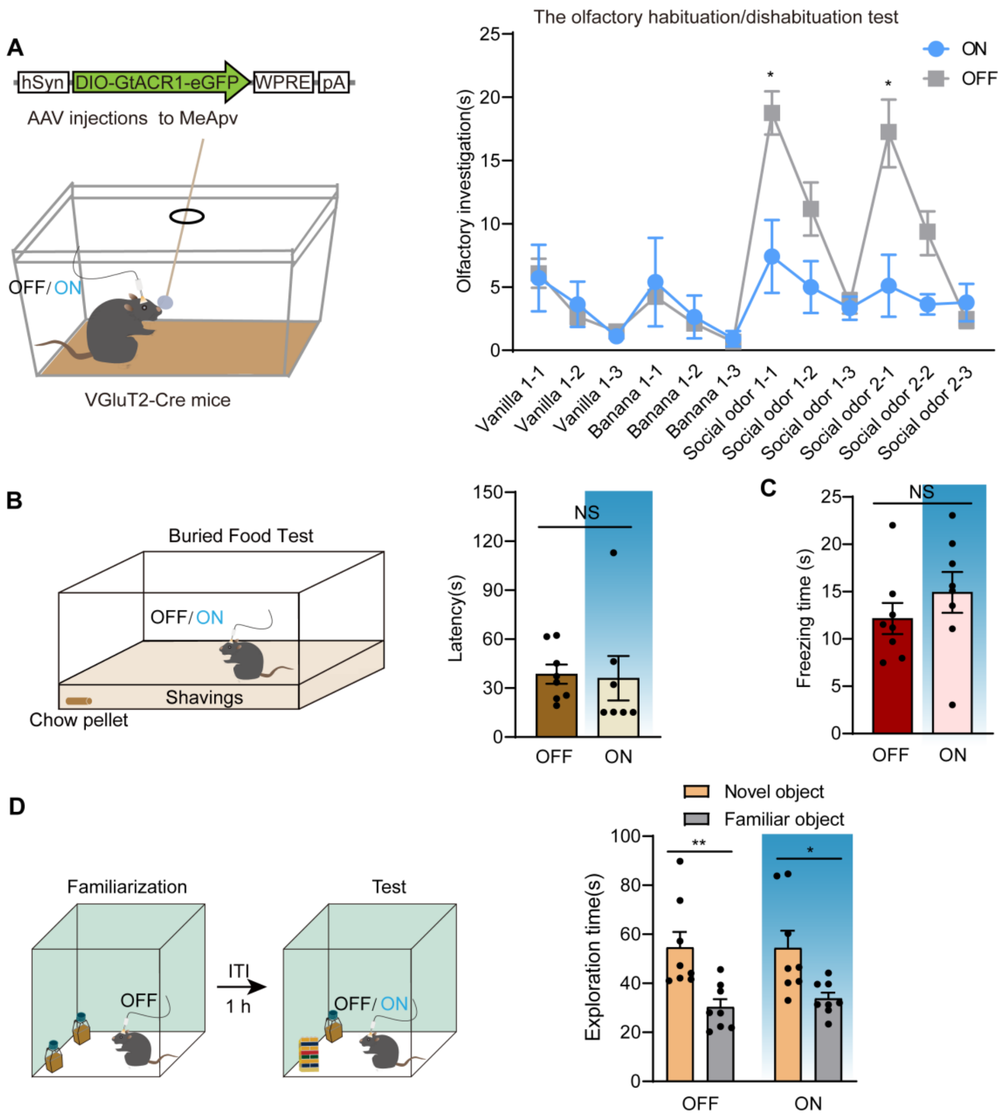

Fig. S4. Inhibition of MeApv VGluT2 neurons impairs neither general olfactory nor novelty-seeking behavior (related to Figure 2).

(**A**) The olfactory habituation-dishabituation test (n = 8; Social odor 1-1, P = 0.0391; Social odor 2-1, P = 0.0156; Wilcoxon matched-pairs signed-rank test).

(**B**) Buried food test (n = 8; P = 0.2319, Mann-Whitney test).

(**C**) Predator odor-induced freezing test (n = 8; P = 0.3125, Wilcoxon matched-pairs signed-rank test).

(**D**) Novel object recognition test (n = 8; OFF: P = 0.0078, Wilcoxon matched-pairs signed-rank test; ON: P = 0.0286, paired t-test).

*$P < 0.05$; **$P < 0.01$. NS, not significant. Error bar, SEM.

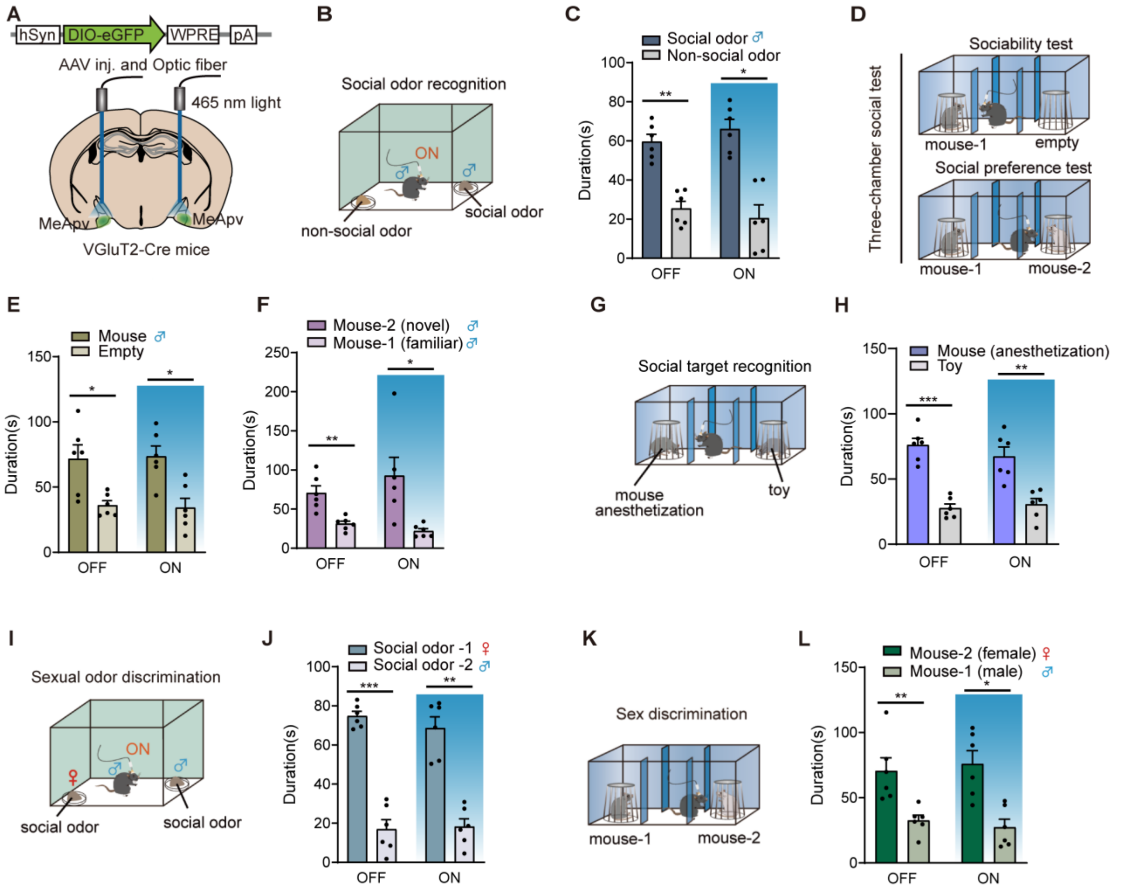

Fig. S5. Control experiments show that GFP expression in MeApv VGluT2 neurons does not alter social behaviors (related to Figure 2).

(**A**) Schematic of viral injection and fiber implantation in MeApv neurons of VGluT2-Cre mice.

(**B**) Social odor recognition test (clean bedding vs. male bedding).

(**C**) Time spent interacting with male bedding versus clean bedding (n = 6; OFF: P = 0.0054; ON: P = 0.0110; paired t-test).

(**D**) Three-chamber social test.

(**E**) Sociability test. Time spent sniffing mouse versus empty chamber (n = 6; OFF: P = 0.0160; ON: P = 0.0241; paired t-test).

(**F**) Social preference test. Time spent interacting with novel versus familiar mouse (n = 6; OFF: P = 0.0057; ON: P = 0.0375; paired t-test).

(**G**) Social target recognition test.

(**H**) Time spent interacting with anesthetized mouse versus toy mouse (n = 6; OFF: P = 0.0004; ON: P = 0.0021; paired t-test).

(**I**) Sexual odor discrimination test (bedding).

(**J**) Time spent interacting with female bedding vs. male bedding (n = 6; OFF: P = 0.0001; ON: P = 0.0017; paired t-test).

(**K**) Sex discrimination test (mouse).

(**L**) Time spent interacting with female mouse versus male mouse (n = 6; OFF: P = 0.0097; ON: P = 0.0257, paired t-test).

*P < 0.05; **P < 0.01; ***P < 0.001. NS, not significant. Error bar, SEM.

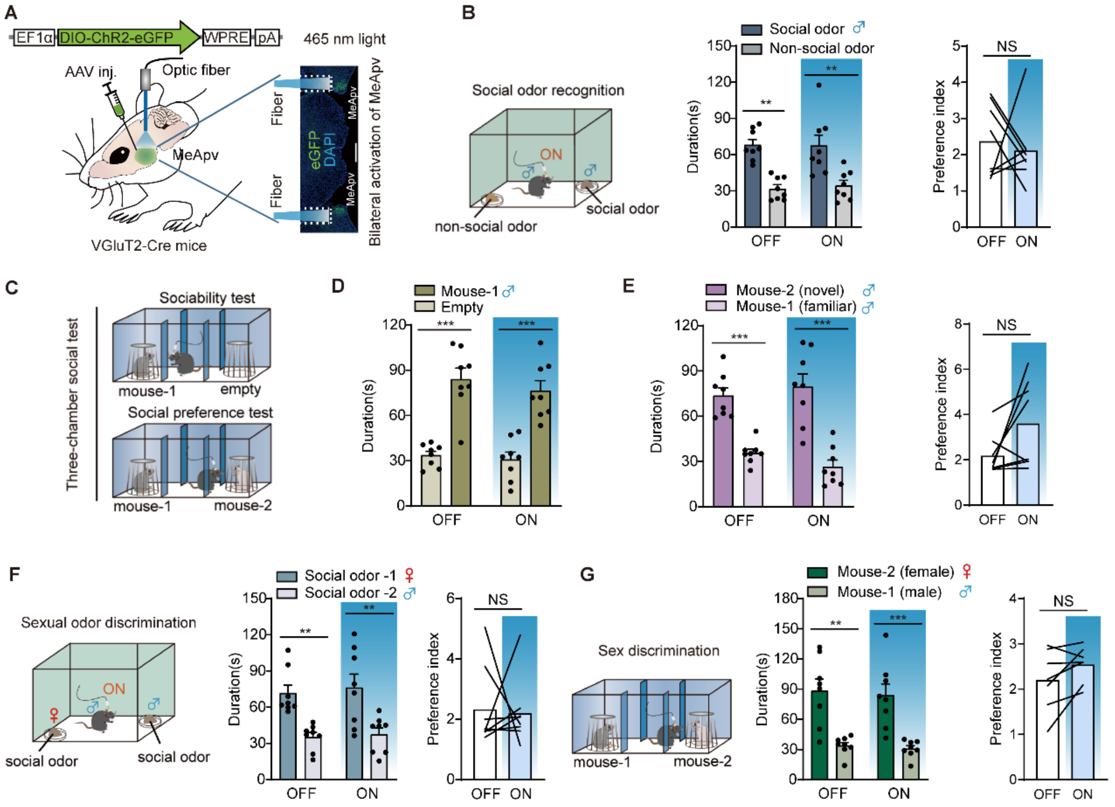

Fig. S6. Optogenetic activation of MeApv VGluT2 neurons does not impair odor-driven social behaviors (related to Figure 2).

(**A**) Viral injection and fiber implantation strategy for optogenetic activation of MeApv neurons in VGluT2-Cre mice. Scale bar, 500 µm.

(**B**) Left: Social odor recognition test (clean bedding vs. male bedding). Mid: Time spent investigating male bedding vs. clean bedding (n = 8; OFF: P = 0.0013; ON: P = 0.0091; paired t-test). Right: Social odor preference, OFF vs. ON groups (n = 8; P = 0.9591, Mann-Whitney test).

(**C**) Schematic of three-chamber social test.

(**D**) Sociability test. Time spent sniffing mouse chamber vs. empty chamber (n = 8; OFF: P = 0.0006; ON: P = 0.0005; paired t-test).

(**E**) Social discrimination test. Left: Time spent interacting with novel mouse vs. familiar mouse (n = 8; OFF: P = 0.0007; ON: P = 0.0008; paired t-test); Right: Social preference, OFF vs. ON groups (n = 8; P = 0.0830, Mann-Whitney-test).

(**F**) Left: Sexual odor discrimination test (bedding). Mid: Time spent investigating female bedding vs. male bedding (n = 8; OFF: P = 0.0078; ON: P = 0.0078; Wilcoxon

matched-pairs signed-rank test). Right: Gender-related social odor preference, OFF vs. ON groups (*n* = 8; *P* = 0.5737, Mann-Whitney test).

(**G**) Left: Sex discrimination test (mouse). Mid: Time spent interacting with female mouse vs. male mouse (*n* = 8; OFF: *P* = 0.0078, Wilcoxon matched-pairs signed-rank test; ON: *P* = 0.0007, paired *t*-test). Right: Gender preference, OFF vs. ON groups (*n* = 8; *P* = 0.5054, Mann-Whitney test).

**P* < 0.05; ***P* < 0.01; ****P* < 0.001. NS, not significant. Error bar, SEM.

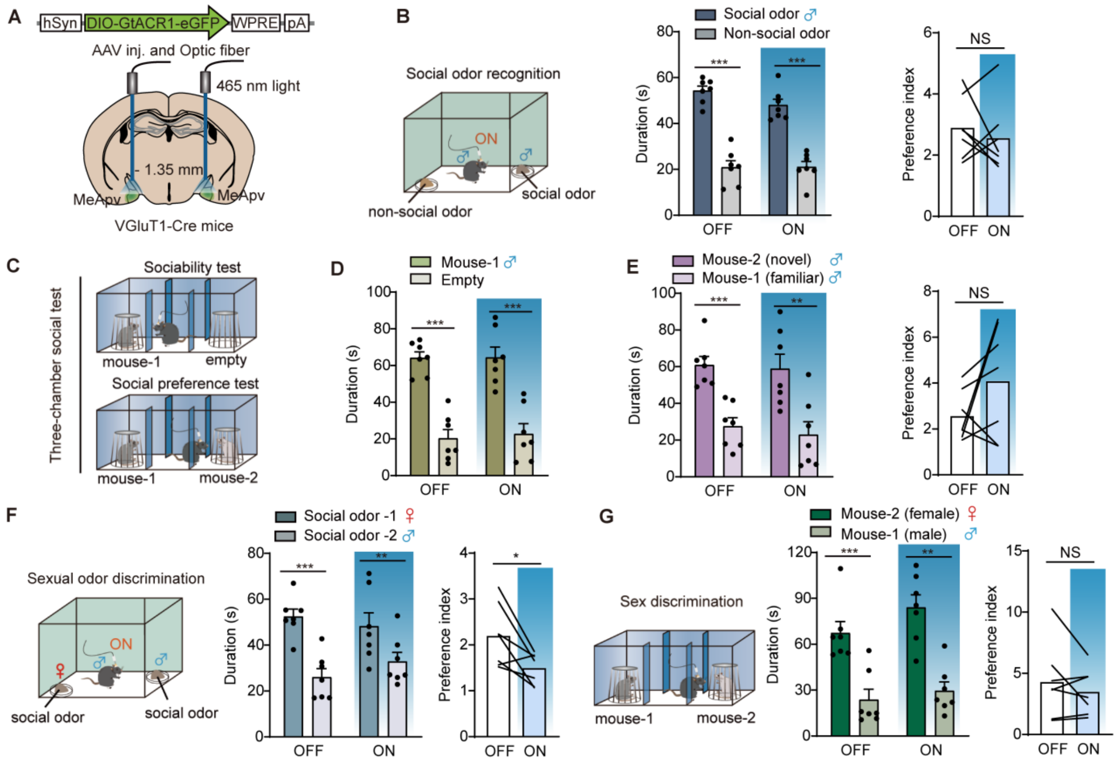

Fig. S7. Optogenetic silencing of MeApv VGluT1 neurons does not impair social recognition (related to Figure 2).

(**A**) Schematic of viral injection and fiber implantation in MeApv neurons of VGluT1-Cre mice.

(**B**) Left: Social odor recognition test (clean bedding vs. male bedding). Mid: Time spent interacting with social odor versus clean bedding (n = 7; OFF: P < 0.0001; ON: P = 0.0002, paired t-test). Right: Social odor preference, OFF vs. ON groups (n = 7; P = 0.5350, Mann-Whitney test).

(**C**) Three-chamber social test apparatus.

(**D**) Sociability test. Time spent sniffing mouse chamber vs. empty chamber (n = 7; OFF: P = 0.0005; ON: P = 0.0009; paired t-test).

(**E**) Social preference test. Left: Time spent interacting with novel mouse vs. familiar mouse (n = 7; OFF: P = 0.0002; ON: P = 0.0025; paired t-test); Right: Social preference, OFF vs. ON groups (n = 7; P = 0.1556, unpaired t-test).

(**F**) Left: Sexual odor discrimination test (bedding). Mid: Time spent investigating female bedding vs. male bedding (n = 7; OFF: P = 0.0001; ON: P = 0.0077; paired t-

test). Right: Gender-related social odor preference, OFF vs. ON groups (n = 7; P = 0.0322, unpaired t-test).

(**G**) Left: Sex discrimination test (mouse). Mid: Time spent interacting with female mouse vs. male mouse (n = 7; OFF: P = 0.0156, paired t-test; ON: P = 0.0023, Wilcoxon matched-pairs signed-rank test). Right: Gender preference, OFF vs. ON groups (n = 7; P = 0.5683, unpaired t-test).

*$P < 0.05$; **$P < 0.01$; ***$P < 0.001$. NS, not significant. Error bar, SEM.

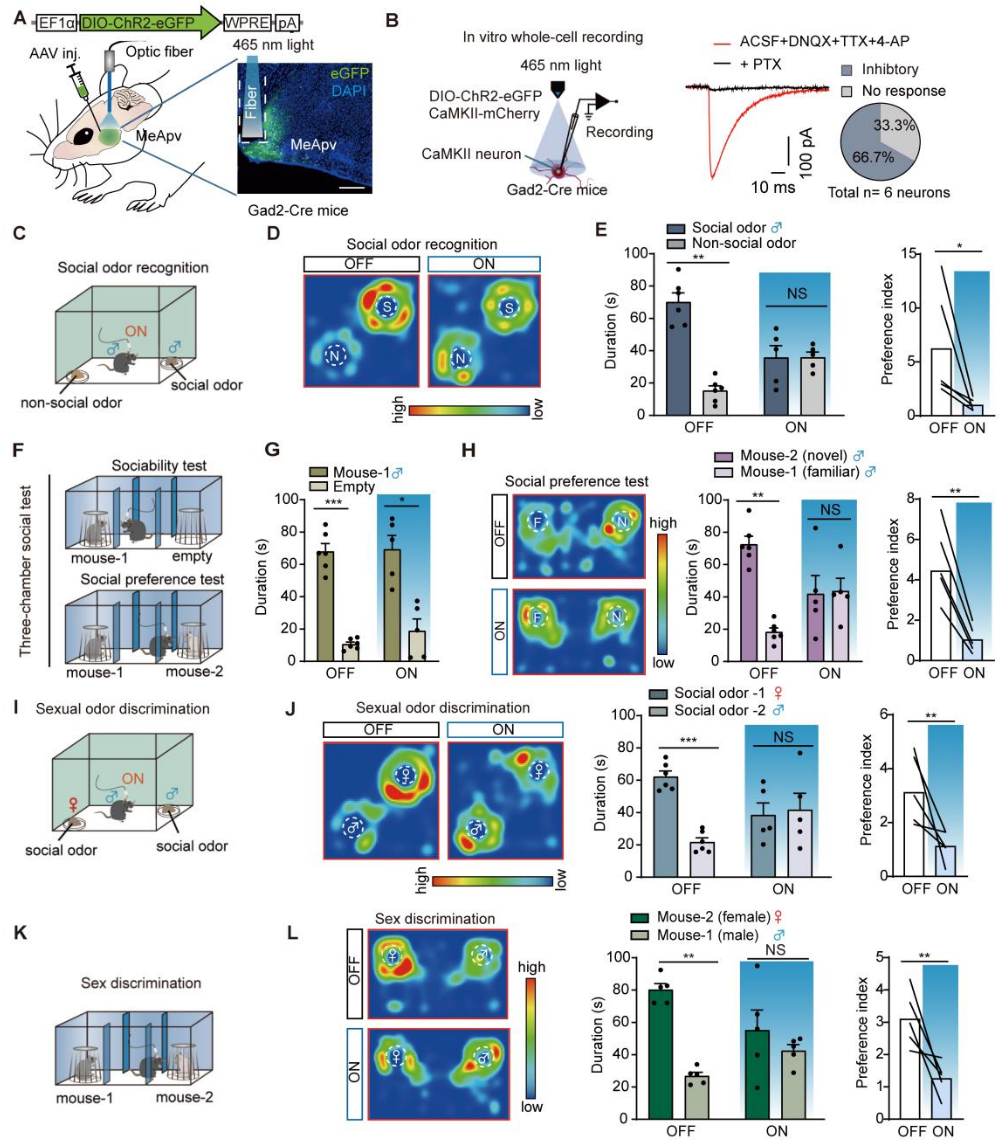

Fig. S8. Optogenetic activation of MeApv Gad2 neurons induces social recognition impairment (related to Figure 2).

(**A**) Viral injection and fiber implantation strategy for MeApv neurons in Gad2-Cre mice. ChR2-expressing neurons were activated by blue light (465 nm, 10 Hz, 5 ms pulses). Scale bar, 250 μm.

(**B**) Whole-cell patch-clamp recording of activated neurons.

(**C**) Social odor recognition test paradigm (clean bedding vs male bedding).

(**D**) Representative heatmaps (N: non-social odor; S: social odor - male bedding).

(**E**) Left: Social odor recognition: Time spent investigating male bedding vs. clean bedding (*n* = 6; OFF: *P* = 0.0010; ON: *P* = 0.9896; paired *t*-test). Right: Social odor preference, OFF vs. ON groups (*n* = 6; *P* = 0.0360, unpaired *t*-test).

(**F**) Three-chamber social test.

(**G**) Sociability test: Time spent sniffing mouse chamber vs. empty chamber (*n* = 6; OFF: *P* < 0.0001; ON: *P* = 0.0141; paired *t*-test).

(**H**) Left: Representative heatmaps (F: familiar mouse; N: novel mouse). Mid: Social preference test: Time spent with novel mouse vs. familiar mouse (*n* = 6; OFF: *P* = 0.0003; ON: *P* = 0.9018; paired *t*-test). Right: Social preference, OFF vs. ON groups (*n* = 6; *P* = 0.0044, unpaired *t*-test).

(**I**) Sexual odor discrimination test (bedding).

(**J**) Left: Representative heatmaps (♀: female bedding; ♂: male bedding). Mid: Time spent investigating female bedding vs. male bedding (*n* = 6; OFF: *P* = 0.0005; ON: *P* = 0.8267; paired *t*-test). Right: Gender-related social odor preference, OFF vs. ON groups (*n* = 6; *P* = 0.0079, unpaired *t*-test).

(**K**) Sex discrimination test (mouse).

(**L**) Left: Representative heatmaps (♀: female mouse; ♂: male mouse). Mid: Time spent interacting with female mouse versus male mouse (*n* = 6; OFF: *P* = 0.0008; ON: *P* = 0.2995; paired *t*-test). Right: Gender preference, OFF vs. ON groups (*n* = 6; *P* = 0.0037, unpaired *t*-test).

* *P* < 0.05; ** *P* < 0.01; *** *P* < 0.001; NS, not significant. Error bar, SEM.

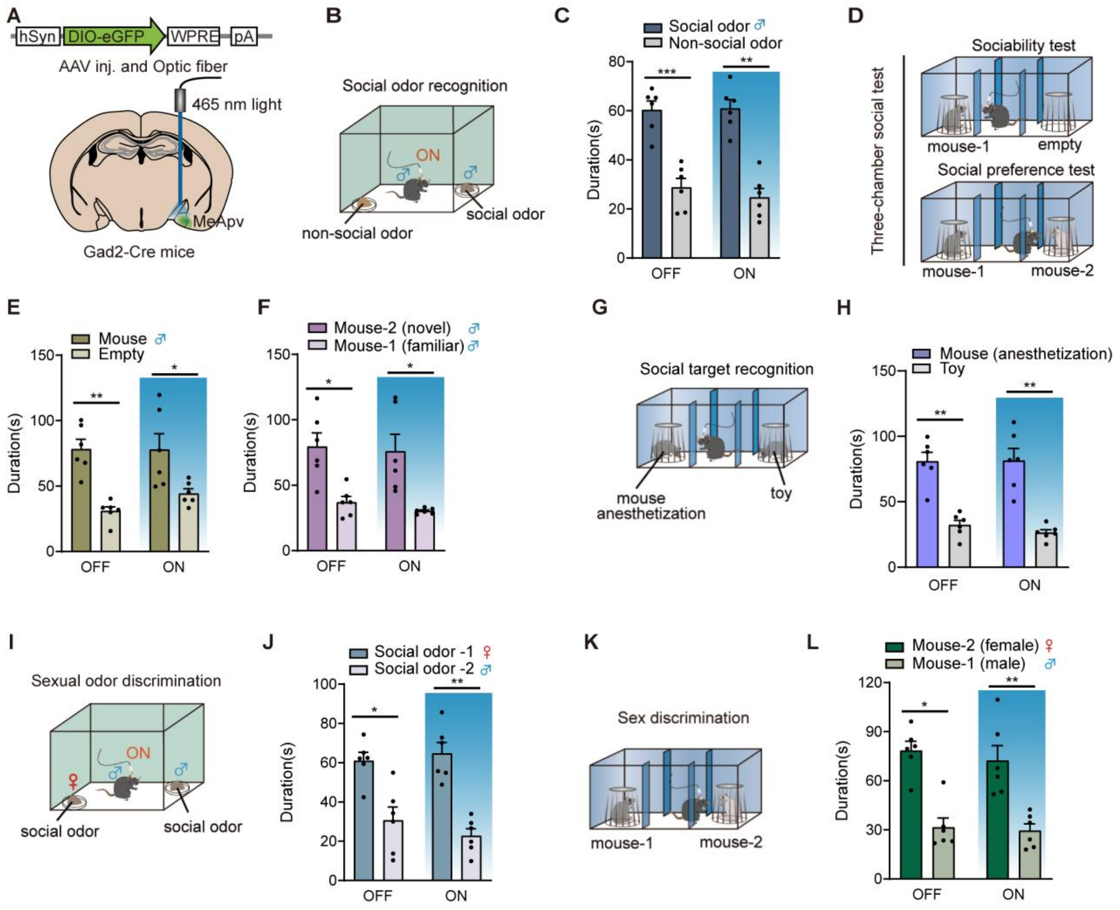

Fig. S9. GFP expression in MeApv Gad2 neurons does not alter social recognition (related to Figure 2).

(**A**) Schematic of viral injection and fiber implantation in MeApv neurons of Gad2-Cre mice.

(**B**) Social odor recognition test (clean bedding vs. male bedding).

(**C**) Time spent interacting with male bedding versus clean bedding (n = 6; OFF: P < 0.0001; ON: P = 0.0031; paired t-test).

(**D**) Three-chamber social test.

(**E**) Sociability test. Time spent sniffing mouse versus empty chamber (n = 6; OFF: P = 0.0039; ON: P = 0.0162; paired t-test).

(**F**) Social preference test. Time spent interacting with novel versus familiar mouse (n = 6; OFF: P = 0.0170; ON: P = 0.0172; paired t-test).

(**G**) Social target recognition test.

(**H**) Time spent interacting with anesthetized mouse versus toy mouse (n = 6; OFF: P = 0.0024; ON: P = 0.0026; paired t-test).

(**I**) Sexual odor discrimination test (bedding).

(**J**) Time spent interacting with female bedding vs. male bedding (n = 6; OFF: P = 0.0131; ON: P = 0.0012; paired t-test).

(**K**) Sex discrimination test (mouse).

(**L**) Time spent interacting with female mouse versus male mouse (n = 6; OFF: P = 0.0313, Wilcoxon matched-pairs signed-rank test; ON: P = 0.0056, paired t-test).

*$P < 0.05$; **$P < 0.01$; ***$P < 0.001$. NS, not significant. Error bar, SEM.

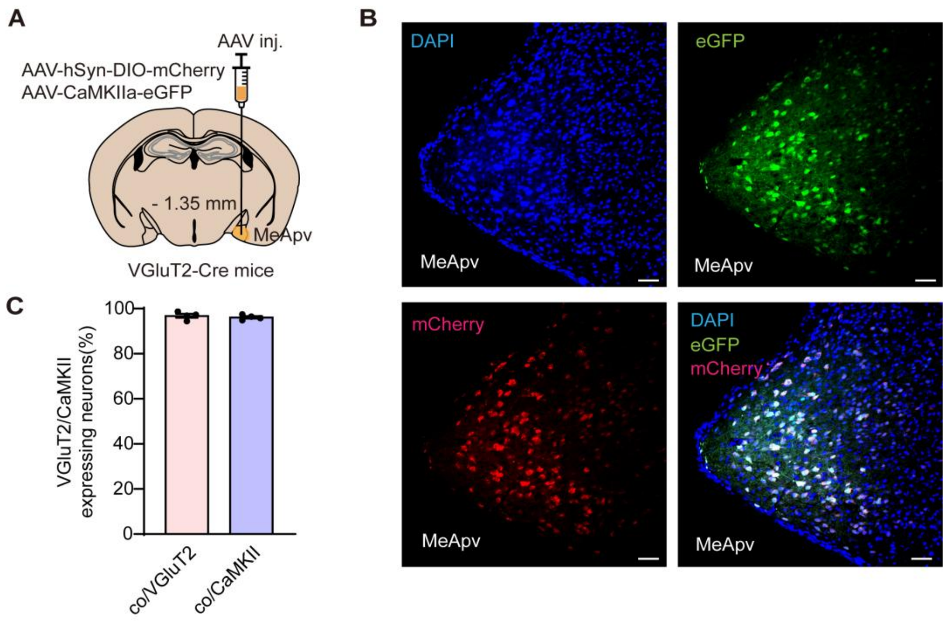

Fig. S10. Co-localization of CaMKIIα and VGluT2 in MeApv neurons.

(**A**) Schematic of the viral injection strategy in the MeApv of VGluT2-Cre mice.

(**B**) Representative images showing viral-meditated fluorescence expression in the MeApv. Scale bars, 50 μm.

(**C**) Analysis of marker overlap. CaMKIIα was expressed in 96.81% of VGluT2^{+} neurons, and VGluT2 was expressed in 96.14% of CaMKIIα^{+} (*n* = 4 mice per group). Error bar, SEM.

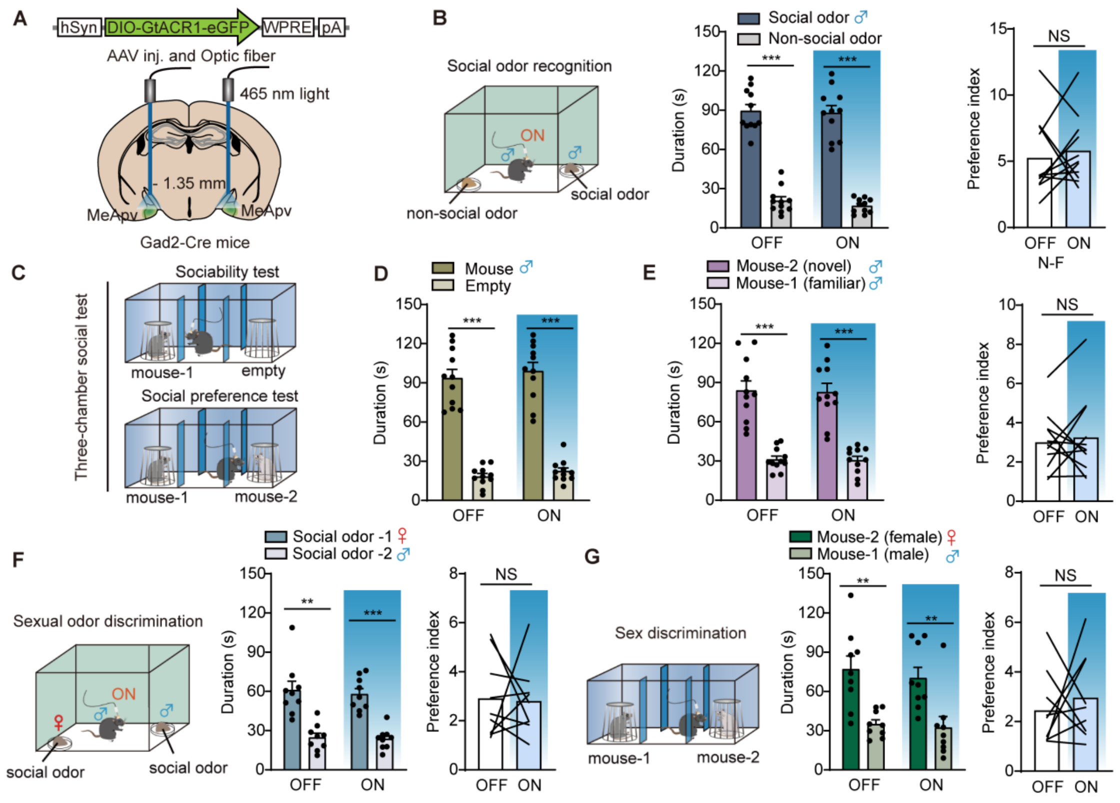

Fig. S11. Optogenetic silencing of MeApv Gad2 neurons does not disrupt social recognition (related to Figure 2).

(**A**) Schematic of viral injection and fiber implantation in MeApv neurons of Gad2-Cre mice.

(**B**) Left: Social odor recognition test (clean bedding vs. male bedding). Mid: Time spent interacting with male bedding versus clean bedding (n = 11; OFF: P < 0.0001; ON: P < 0.0001; paired t-test). Right: Social odor preference, OFF vs. ON groups (n = 11; P = 0.5190, Mann-Whitney test).

(**C**) Three-chamber social test.

(**D**) Sociability test. Time spent sniffing mouse versus empty chamber (n = 11; OFF: P < 0.0001, paired t-test; ON: P = 0.0010, Wilcoxon matched-pairs signed-rank test).

(**E**) Social preference test. Left: Time spent interacting with novel versus familiar mouse (n = 11; OFF: P = 0.0002; ON: P < 0.0001; paired t-test); Right: Social preference, OFF vs. ON groups (n = 11; P > 0.9999, Mann-Whitney test).

(**F**) Left: Sexual odor discrimination test (bedding). Mid: Time spent interacting with female bedding vs. male bedding (n = 9; OFF: P = 0.0039, Wilcoxon matched-pairs

signed-rank test; ON: P = 0.0004, paired t-test). Right: Gender-related social odor preference, OFF vs. ON groups (n = 9; P = 0.8806, unpaired t-test).

(**G**) Left: Sex discrimination test (mouse). Mid: Time spent interacting with female mouse versus male mouse (n = 9; OFF: P = 0.0062, paired t-test; ON: P = 0.0039, Wilcoxon matched-pairs signed-rank test). Right: Gender preference, OFF vs. ON groups (n = 9; P = 0.5154, unpaired t-test).

**$P < 0.01$; ***$P < 0.001$. NS, not significant. Error bar, SEM.

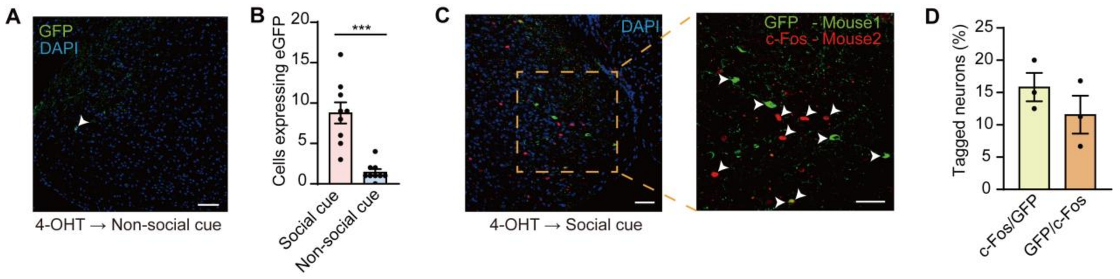

Fig. S12. The specificity of the ESARE system (related to Figure 5).

(**A**) Representative images of ESARE GFP expression (non-social cue) in the MeApv. Scale bars, 50 μm.

(**B**) ESARE GFP expression of social cue (related to Fig.5E) versus non-social cue, 9 slices from 3 mice, $P < 0.001$, Mann-Whitney test.

(**C**) Representative images of c-Fos-positive (labeled Mouse-2) and ESARE GFP expression (labeled Mouse-1) in MeApv. Scale bars, 50 μm.

(**D**) Overlap of c-Fos and GFP.

***$P < 0.001$. Error bar, SEM.

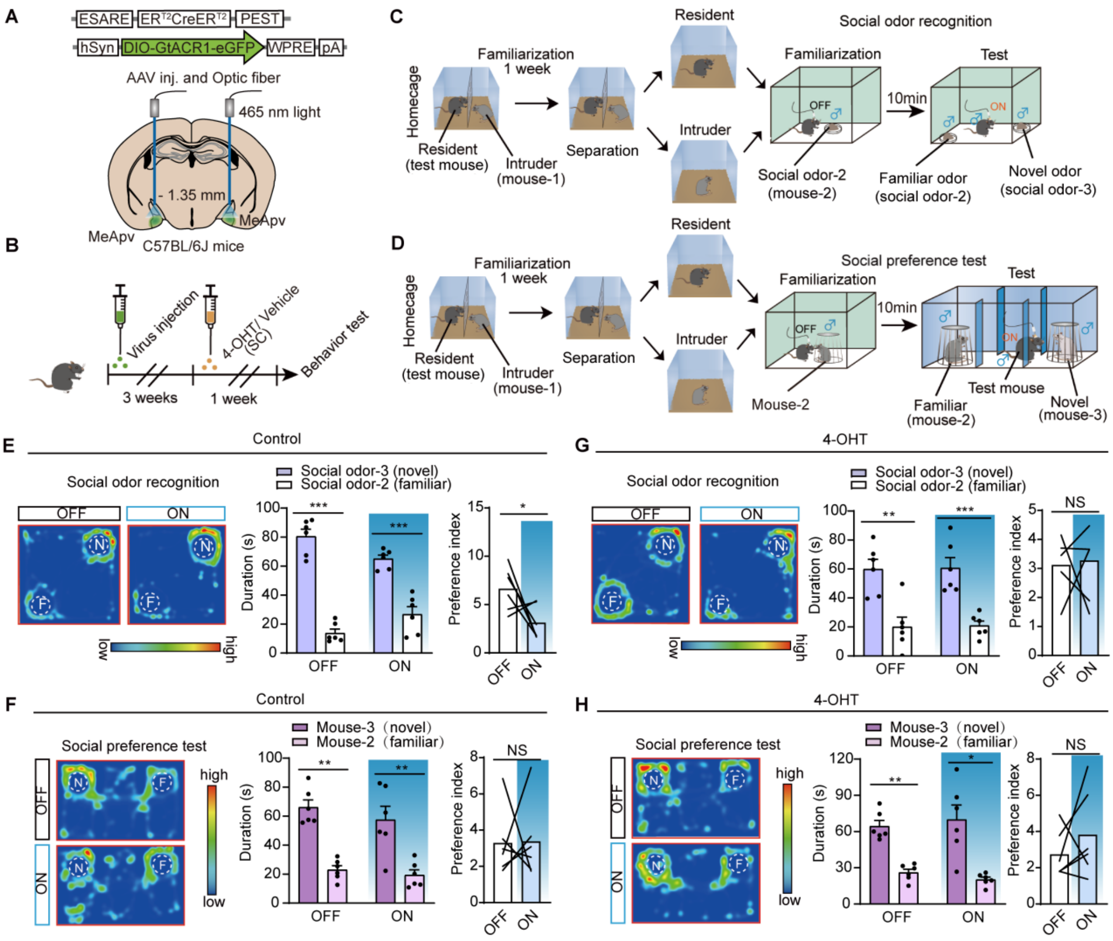

Fig. S13. Inhibition of specific VGluT2 neurons in the MeApv does not impair general social odor recognition (related to Figure 5).

(**A**) Schematic of viral injection and fiber implantation in MeApv neurons of C57BL/6J mice.

(**B**) Experimental design schematic.

(**C**) Social odor recognition test paradigm.

(**D**) Social preference test paradigm.

(**E**) Control groups: (Left) Representative investigation heatmaps. (Mid) Time spent investigating odors from Mouse 2 (familiar) versus Mouse 3 (novel) (n = 6; OFF: P = 0.0313, Wilcoxon matched-pairs signed-rank test; ON: P = 0.0008; paired t-test). (Right) Social odor preference, OFF vs. ON groups (n = 6; P = 0.0260, Mann-Whitney test).

(**F**) Control groups: (Left) Representative interaction heatmaps. (Mid) Time spent with Mouse 2 versus Mouse 3 (*n* = 6; OFF: *P* = 0.0020; ON: *P* = 0.0070; paired *t*-test). (Right) Social preference, OFF vs. ON groups (*n* = 6; *P* = 0.9426, unpaired *t*-test).

(**G**) 4-OHT groups: (Left) Representative investigation heatmaps. (Mid) Time spent with odors of Mouse 2 (familiar) versus Mouse 3 (novel) (*n* = 6; OFF: *P* = 0.0011; ON: *P* = 0.0043; paired *t*-test). (Right) Social odor preference, OFF vs. ON groups (*n* = 6; *P* = 0.8367, unpaired *t*-test).

(**H**) 4-OHT groups: (Left) Representative interaction heatmaps. (Mid) Time spent with Mouse 2 versus Mouse 3 (*n* = 6; OFF: *P* = 0.0029; ON: *P* = 0.0196; paired *t*-test). (Right) Social preference, OFF vs. ON groups (*n* = 6; *P* = 0.3570, unpaired *t*-test).

**P* < 0.05; ***P* < 0.01; ****P* < 0.001. NS, not significant. Error bar, SEM.

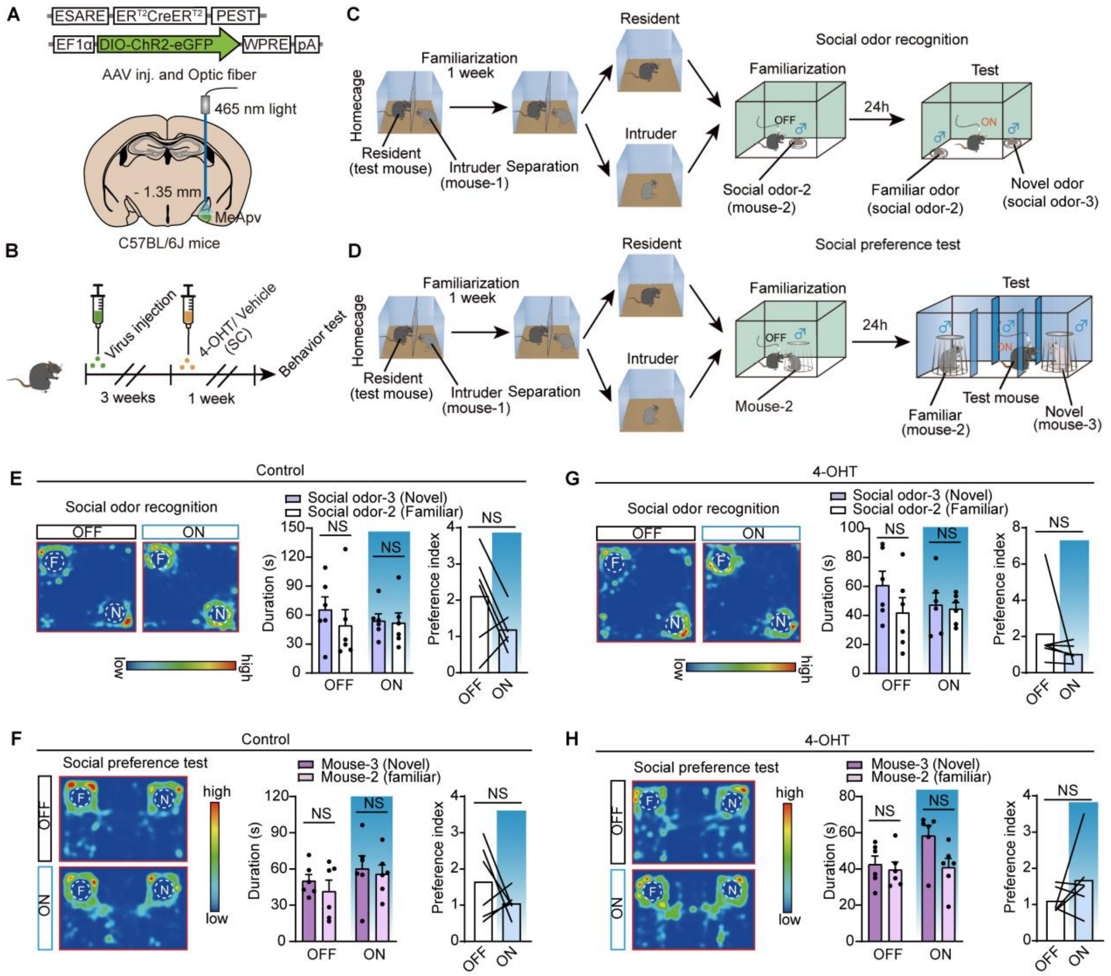

Fig. S14. Activation of specific VGluT2 neurons in MeApv fails to enhance general social odor recognition (related to Figure 6).

(**A**) Viral injection and fiber implantation schematic in MeApv neurons of C57BL/6J mice.

(**B**) Experimental design schematic.

(**C**) Social odor recognition test paradigm (familiar vs. novel odors).

(**D**) Social preference test paradigm (familiar vs. novel mice).

(**E**) Control groups: (Left) Representative investigation heatmaps. (Mid) Time spent investigating familiar vs. novel social odors (n = 6; OFF: P = 0.5625, Wilcoxon matched-pairs signed-rank test; ON: P = 0.8011, paired t-test). (Right) Social odor preference, OFF vs. ON groups (n = 6; P = 0.1445, unpaired t-test).

(**F**) Control groups: (Left) Representative interaction heatmaps. (Mid) Time spent with familiar mouse vs. novel mouse (n = 6; OFF: P = 0.5073; ON: P = 0.4578; paired t-test). (Right) Social preference, OFF vs. ON groups (n = 6; P = 0.2069, unpaired t-test).

(**G**) 4-OHT groups: (Left) Representative investigation heatmaps. (Mid) Time spent investigating with familiar odor vs. novel social odors (*n* = 6; OFF: *P* = 0.2460; ON: *P* = 0.9764; paired *t*-test). (Right) Social odor preference, OFF vs. ON groups (*n* = 6; *P* = 0.1797, Mann-Whitney test).

(**H**) 4-OHT groups: (Left) Representative interaction heatmaps. (Mid) Time spent with familiar mouse vs. novel mouse (*n* = 6; OFF: *P* = 0.5640, paired *t*-test; ON: *P* = 0.2188, Wilcoxon matched-pairs signed-rank test). (Right) Social preference, OFF vs. ON groups (*n* = 6; *P* = 0.2403, Mann-Whitney test).

NS, not significant. Error bar, SEM.

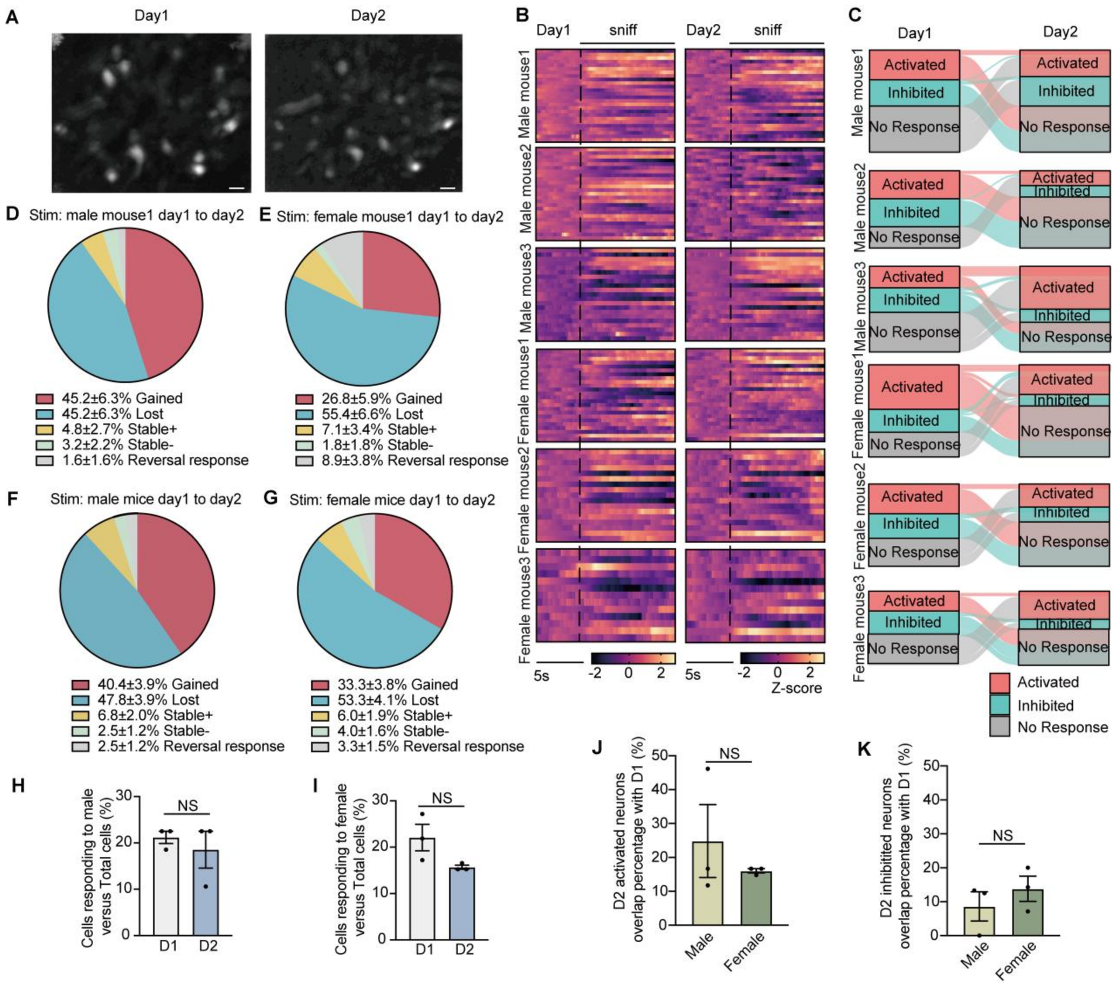

Fig. S15. Longitudinal analysis of neuronal responses to the same individual stimulus across days (related to Figure 4).

(**A**) Representative raw Ca^{2+}imaging frames acquired through a GRIN lens from the same animal in day1 and 2; scale bar, 20 μm.

(**B**) Heatmaps depicting the mean Ca^{2+}activity (z-score) of individual neurons in response to male or female mouse stimuli on days 1 and 2. Neuron identities were consistent across sessions, allowing comparison of stimulus-evoked responses over time. Neurons are aligned across days.

(**C**) Alluvial plot illustrating changes in neuronal responsiveness across days 1 and 2 in response to male or female mouse stimuli. Neurons were classified as activated, inhibited, or nonresponsive; cells inactive on both days were excluded. Each column corresponds to one day, and individual lines represent neurons tracked across sessions. Colors indicate the response category of each neuron on the corresponding day.

(**D** to **G**) Pie charts depicting the proportion of neurons that gained, lost, reversed their responses (from inhibited to activated, or vice versa), or remained stable across days 1 and 2 (D) male mouse1 stimuli, (E) female mouse1 stimuli, (F) male mice summary stimuli, (G) female mice summary stimuli (mean ± SEM). D-E display representative pie chart from individual male mouse 1 and female mouse 1, while F-G present summary data from three mice.
(**H** and **I**) Quantification of the proportion of neurons responding to stimuli in male (H) and female (I) mice on day 1 and day 2. For each sex, data were first calculated separately for each individual mouse (#1–3) and then averaged across mice. (n = 3 mice ; male mice: P > 0.9999; female mice: P = 0.2500; Wilcoxon signed-rank test.
(**J** and **K**) Proportion of neurons activated (J) or inhibited (K) on day1 that remained activated or inhibited on day 2 (n = 3 mice; Activated: P > 0.9999; Inhibited: P = 0.4000; Mann-Whitney test. NS, not significant. Error bar, SEM.

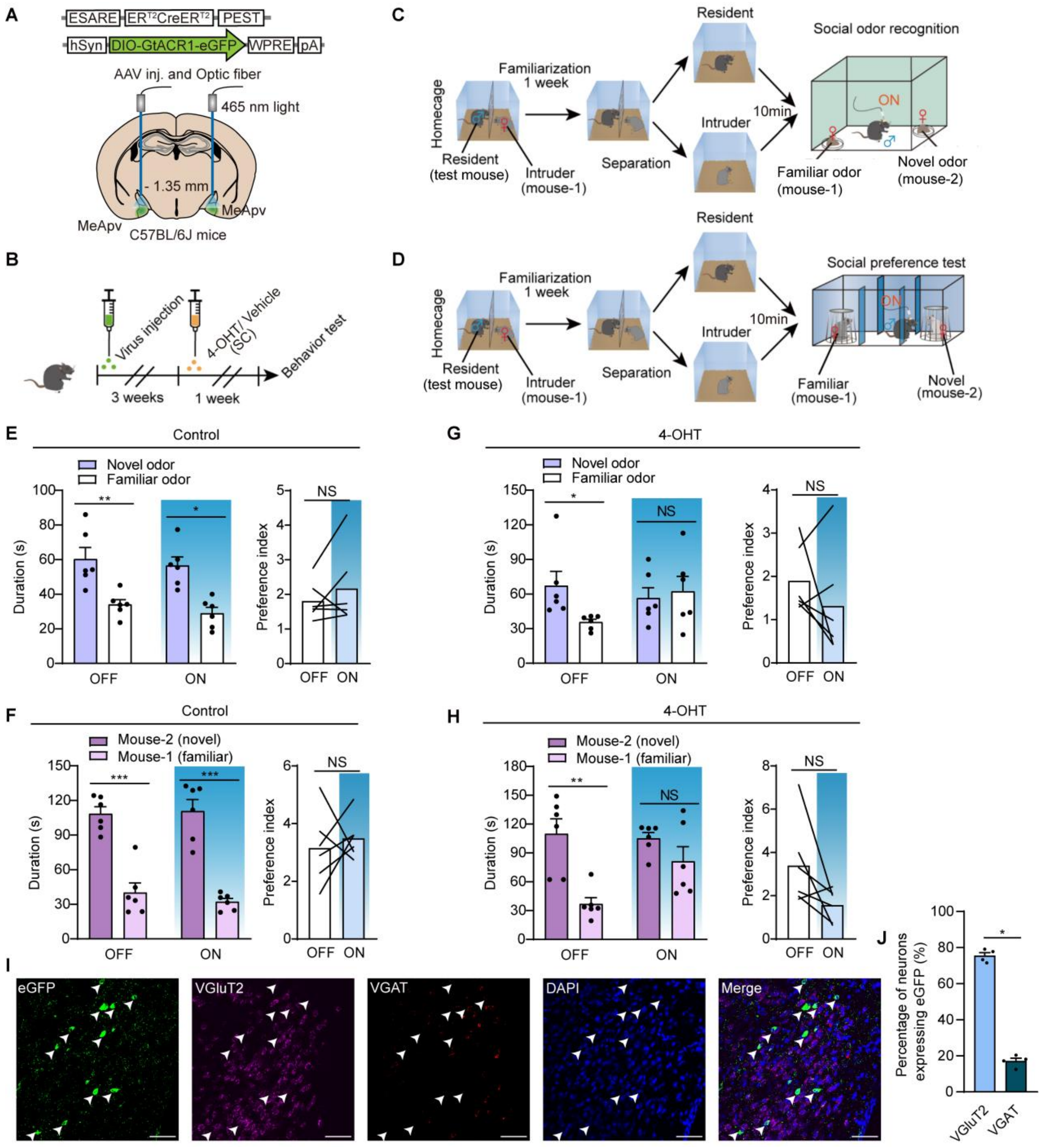

Fig. S16. Inhibition of specific VGluT2 neurons in MeApv impairs recognition for female conspecifics (related to Figure 5).

(**A**) Viral injection and fiber implantation schematic in MeApv neurons of C57BL/6J mice.

(**B**) Experimental design schematic.

(**C**) Social odor recognition test paradigm.

(**D**) Social preference test paradigm.

(**E**) Social odor recognition. Left (Control): Time investigating odors of female Mouse 1 vs female Mouse 2 (n = 6; OFF: P = 0.0100; ON: P = 0.0119; paired t-test); Right: Social odor preference, OFF vs. ON groups (n = 6; P = 0.9372, Mann-Whitney test).

(**F**) Social recognition. Left (Control): Time with female Mouse 1 vs female Mouse 2 (*n* = 6; OFF: *P* = 0.0003; ON: *P* = 0.0002; paired *t*-test); Right: Social preference, OFF vs. ON groups (*n* = 6; *P* = 0.5840, unpaired *t*-test).

(**G**) Social odor recognition. Left (4-OHT): Time investigatingspent with odors of female Mouse 1 vs female Mouse 2 (*n* = 6; OFF: *P* = 0.0313, Wilcoxon matched-pairs signed-rank test; ON: *P* = 0.7760, paired *t*-test); Right: Social odor preference, OFF vs. ON groups (*n* = 6; *P* = 0.2403, Mann-Whitney test).

(**H**) Social recognition. Left (4-OHT): Time with female Mouse 1 vs female Mouse 2 (*n* = 6; OFF: *P* = 0.0044; ON: *P* = 0.2945; paired *t*-test); Right: Social preference, OFF vs. ON groups (*n* = 6; *P* = 0.0931, Mann-Whitney test).

(**I**) RNAscope validation showing VGluT2 co-localization in labeled neurons. Scale bars, 50 μm.

(**J**) Quantification of $eGFP^+$ cells (*n* = 4 mice, *P* = 0.0286, Mann-Whitney test).

**P* < 0.05; ***P* < 0.01; ****P* < 0.001; NS, not significant. Error bar, SEM.

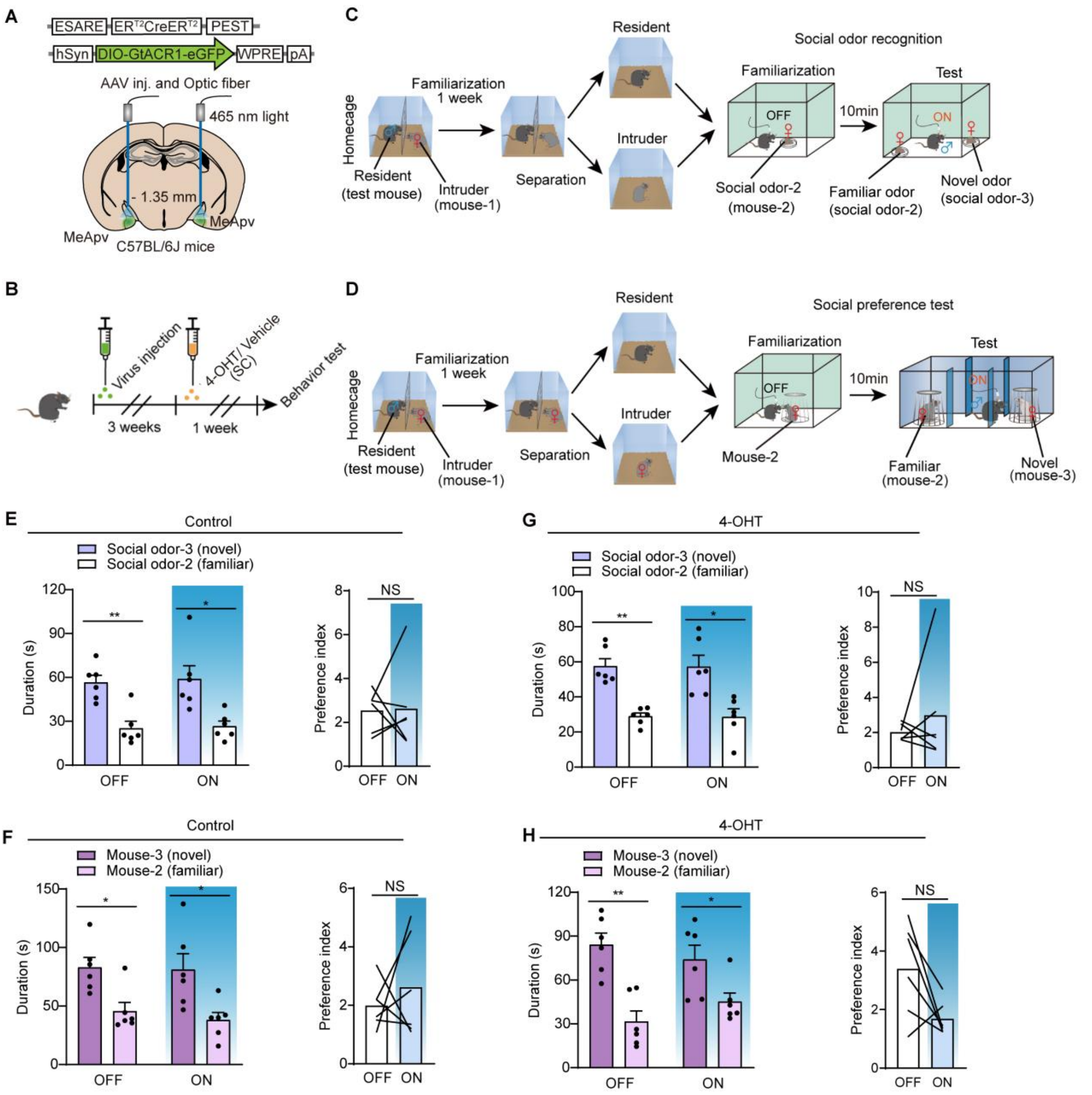

Fig. S17. Inhibition of specific VGluT2 neurons in MeApv does not impair general recognition of female conspecifics (related to Figure 5).

(**A**) Viral injection and fiber implantation schematic in MeApv neurons of C57BL/6J mice.

(**B**) Experimental design schematic.

(**C**) Social odor recognition test paradigm.

(**D**) Social preference test paradigm.

(**E**) Social odor recognition. Left (Control): Time investigating odors of female Mouse 2 vs female Mouse 3 (n = 6; OFF: P = 0.0313, Wilcoxon matched-pairs signed-rank test; ON: P = 0.0012; paired t-test); Right: Social odor preference, OFF vs. ON groups (n = 6; P = 0.3939, Mann-Whitney test).

(**F**) Social recognition. Left (Control): Interacting time with female Mouse 2 vs female Mouse 3 (*n* = 6; OFF: *P* = 0.0313, Wilcoxon matched-pairs signed-rank test; ON: *P* = 0.0476, paired *t*-test); Right: Social preference, OFF vs. ON groups (*n* = 6; *P* = 0.4419, unpaired *t*-test).

(**G**) Social odor recognition. Left (4-OHT): Time investigating odors of female Mouse 2 vs female Mouse 3 (*n* = 6; OFF: *P* = 0.0409; ON: *P* = 0.0428; paired *t*-test); Right: Social odor preference, OFF vs. ON groups (*n* = 6; *P* > 0.9999, Mann-Whitney test).

(**H**) Social recognition. Left (4-OHT): Interacting time with female Mouse 2 vs female Mouse 3 (*n* = 6; OFF: *P* = 0.0044, paired *t*-test; ON: *P* = 0.0313, Wilcoxon matched-pairs signed-rank test); Right: Social preference, OFF vs. ON groups (*n* = 6; *P* = 0.1320, Mann-Whitney test).

*P<0.05; **P<0.01. NS, not significant. Error bar, SEM.
